# AI-driven label-free Raman spectromics for intraoperative spinal tumor assessment

**DOI:** 10.1038/s41746-025-02279-6

**Published:** 2026-03-17

**Authors:** David Reinecke, Nina Müller, Anna-Katharina Meissner, Gina Fürtjes, Lili Leyer, Claire Wang, Adrian Ion-Margineanu, Nader Maarouf, Andrew Smith, Todd C. Hollon, Cheng Jiang, Xinhai Hou, Abdulkader Al-Shughri, Lisa I. Körner, Georg Widhalm, Thomas Roetzer-Pejrimovsky, Matija Snuderl, Sandra Camelo-Piragua, John G. Golfinos, Roland Goldbrunner, Daniel A. Orringer, Niklas von Spreckelsen, Volker Neuschmelting

**Affiliations:** 1 Department of General Neurosurgery, Center for Neurosurgery, Faculty of Medicine and University Hospital Cologne, University of Cologne, Cologne, Germany; 2 Department of Neurosurgery, New York University Grossman School of Medicine, New York, NY, USA; 3 Center for Integrated Oncology, Faculty of Medicine and University Hospital Cologne, University of Cologne, Cologne, Germany; 4 Invenio Imaging Inc, Santa Clara, CA, USA; 5 Machine Learning in Neurosurgery Laboratory, Department of Neurosurgery, University of Michigan, Ann Arbor, MI, USA; 6 Institute for Neuropathology, Faculty of Medicine and University Hospital Cologne, University of Cologne, Cologne, Germany; 7 Department of Neurosurgery, Medical University of Vienna, Vienna, Austria; 8 Division of Neuropathology and Neurochemistry, Department of Neurology, Medical University of Vienna, Vienna, Austria; 9 Comprehensive Center for Clinical Neurosciences & Mental Health, Medical University of Vienna, Vienna, Austria; 10 Department of Pathology, New York Grossman School of Medicine, New York, NY, USA; 11 Department of Pathology, University of Michigan, Ann Arbor, MI, USA; 12 Department of Neurosurgery, WKK Westküstenkliniken Heide, Heide, Germany

**Keywords:** Cancer, Medical research, Oncology

## Abstract

Spinal tumor surgery requires rapid tissue diagnosis to guide surgical decisions and further treatment strategies, yet current intraoperative methods are time-intensive and require specialized expertise. No AI systems exist for real-time spinal tumor classification during surgery. We developed SpineXtract, the first AI-powered system for rapid intraoperative spinal tumor diagnosis using stimulated Raman histology (SRH) — a label-free Raman spectromics imaging technique without tissue processing available during surgery. We created a transformer-based classifier optimized for spinal tissue characteristics to identify common tumor types: meningioma, schwannoma, ependymoma, and metastasis. The system was tested in an international, multicenter, simulated, single-arm study using existing SRH datasets (44 patients, 142 slide-images) from three international institutions, with final pathological diagnosis as reference standard. SpineXtract achieved a 92.9% macro-average balanced accuracy (95% CI: 85.5–98.2) within 5 minutes (tumor-specific accuracy range, 84.2–98.6%), while providing quantitative microscopic feedback for granular tissue analysis. Performance remained consistent across institutions (macro balanced accuracy 91.4–92.0%) and outperformed existing brain tumor classifiers by 15.6%. Our results demonstrate clinical applicability, enabling rapid intraoperative diagnosis with performance exceeding current methods, potentially transforming intraoperative diagnostic workflows in spinal tumor surgery.

## Introduction

Intraoperative diagnosis plays an essential role in the management of spinal tumors. Metastases, meningioma, schwannomas, and ependymomas are among the most common types, and magnetic resonance imaging (MRI) is considered the diagnostic goldstandard^1–3^. However, the suspected diagnosis often remains unclear despite MRI. In many cases, MRI diagnostics may be contraindicated, and often, regardless of the preoperative imaging, the tumor cannot be clearly classified based on its intraoperative macroscopic appearance. The definitive diagnosis depends on histopathological evaluation, which can take multiple days following biopsy collection. A prolonged waiting period can delay further diagnostics and treatment, as well as risking clinical harm in case of space-occupying lesions and nerve compression. In such situations, accurate intraoperative diagnosis is essential for clinical decision-making. To address this, surgeons currently rely on rapid frozen section diagnostics. While this method can provide valuable information, it is limited by several factors, including a time-consuming pathological workforce (up to 30 minutes and more), only covered by specialists during regular working hours^4^. Furthermore, the reported diagnostic accuracy of rapid frozen section in spinal tumors varies between 86.6-88.6%^5–7^. Stimulated Raman Histology (SRH) is an evolving technology in digital pathology and has recently been introduced as rapid and precise non-inferior alternative to rapid frozen section workflow^8,9^. This label-free optical imaging technique, based on Raman spectromics, generates high-resolution, virtual hematoxylin and eosin (H&E)-like images without the need for chemical fixation or staining. The resulting images can be assessed either by a pathologist or analyzed in near-real-time by artificial intelligence (AI), facilitating fast and reliable human reviewer-independent intraoperative diagnostic decision-making. Current diagnostic AI-based central nervous system (CNS) tumor classifiers demonstrate diagnostic accuracy comparable to that of rapid frozen section analysis, and even enable molecular pathology-integrated diagnosis^10–15^. Although various CNS tumor algorithms have already been employed, no specialized AI classifier has been developed for spinal tumors. In this international multicenter simulated single-arm study, we evaluated the performance of an existing SRH-CNS tumor classification model on spinal tumors and subsequently developed an anatomical site-specific algorithm designed to classify the four most common types of spinal tumors.

## Results

### Development and multicentric testing of SpineXtract

**Fig. 1:**
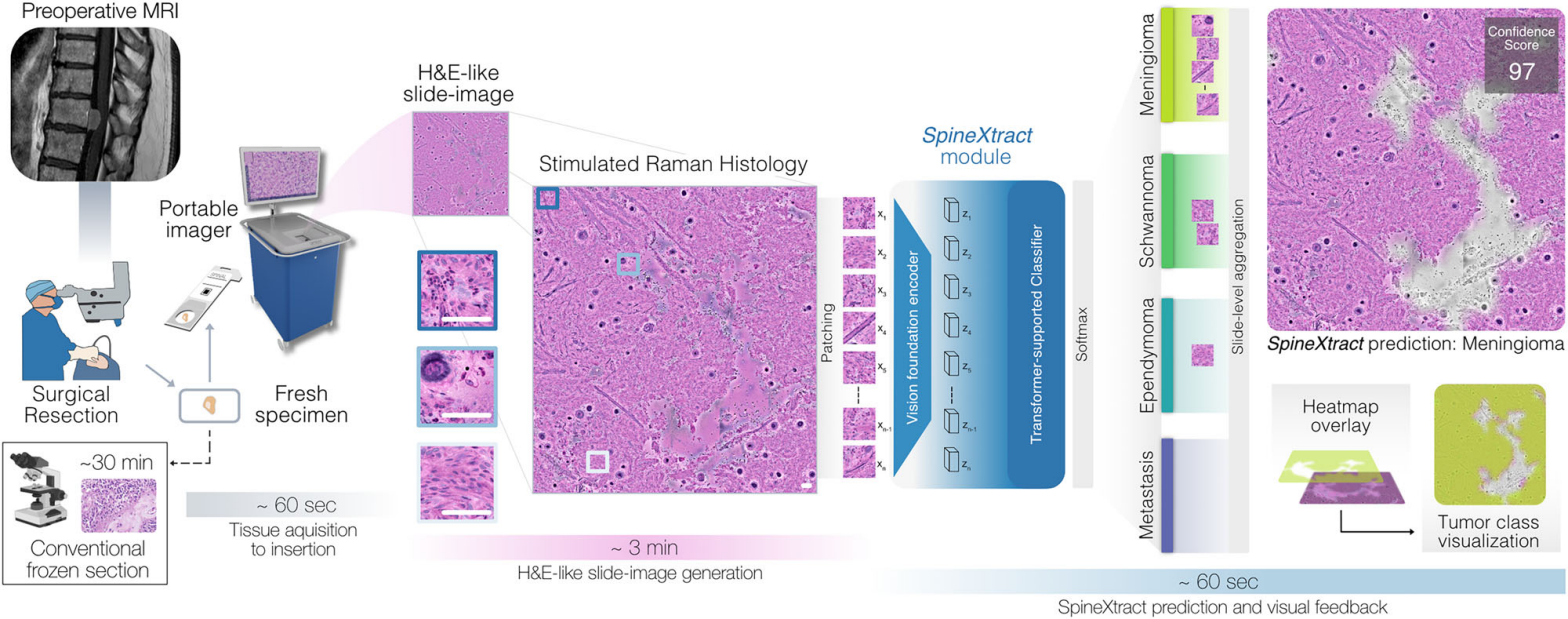
Clinical workflow of SpineXtract for intraoperative spine tumor diagnosis. Beginning with preoperative MRI for surgical planning, the process continues through surgical resection where a fresh unprocessed tissue specimen is obtained. This specimen is squeezed onto a slide and forwarded into a portable stimulated Raman histology (SRH) imager that generates label-free digital H&E-like slide-images. The SpineXtract module then processes patch through vision encoder and transformer-based classification components, analyzing the diagnostic features to classify the specimen among four tumor types: meningioma, schwannoma, ependymoma, or metastasis. The output includes an aggregated confidence score (shown as 97 for the meningioma example) on image-level and visualization tools including heatmap overlays and tumor class visualization (yellow for meningioma class). The result provides the surgeons and surgical pathologists with near real-time histopathological guidance within 5 minutes in total, irrespective of regular working hours and without requiring conventional tissue processing steps.

We developed SpineXtract, the first specified intraoperative visual diagnostic platform designed to differentiate among four distinct spinal neoplasm types through label-free SRH imaging of freshly, unprocessed tissue specimens (Fig. 1). This system employs a fully automated artificial intelligence pipeline enabling rapid intraoperative assessment of SRH slide images acquired during spinal tumor surgery. SpineXtract’s pipeline incorporates: our proprietary self-supervised learning framework to develop a robust foundation vision encoder for SRH image patches, a novel customized transformer-patch-based classification mechanism to distinguish between meningioma, schwannoma, ependymoma and metastasis, and an advanced semantic probability segmentation approach generating interpretable visual heatmaps for intraoperative utilization by surgeons and pathologists. (See Supplementary Fig. 3-5).

We validated SpineXtract in an international multicenter simulated single-arm trial to assess its generalizability across different medical institutions, patient characteristics, and WHO spinal tumor classifications. Validation testing was implemented across three academic tertiary medical institutions: NYU, UM, and MUV. The cohort comprised 142 individual SRH images from 44 patients for evaluation. No adverse events related to tissue sampling for SRH imaging were observed, as all specimens were obtained as part of standard surgical procedures.

**Fig. 2:**
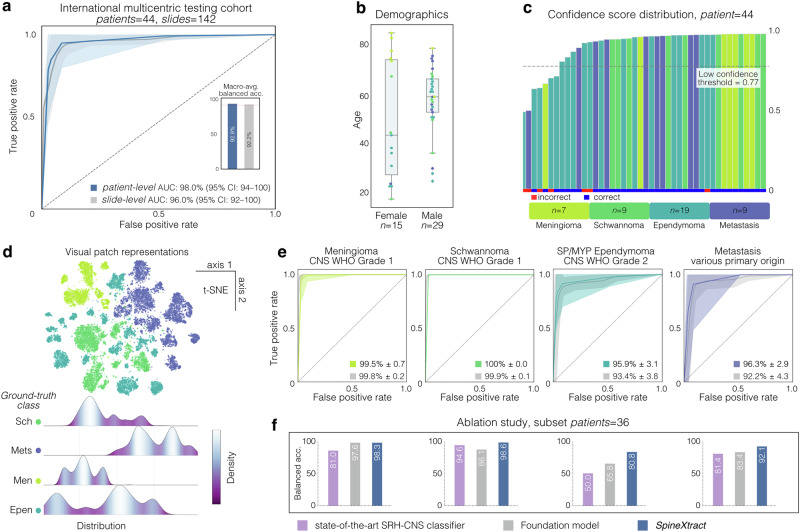
Comprehensive performance evaluation of SpineXtract in an international multicentric testing cohort. **a** Displays the ROC curve analysis of the international testing cohort (patients=44), demonstrating SpineXtract’s strong performance with inset showing mean balanced accuracy of 92.9%. **b** The demographic distribution of the testing cohort by sex and age. **c** Illustrates the confidence score distribution of calibrated and aggregated probabilities across all 44 patients, color-coded by tumor type and prediction correctness, showing high confidence scores correlating with correct predictions. Low confidence under threshold indicates resampling or -imaging. **d** Presents t-SNE visualization of patch representations with clear clustering by tumor type (color-coded) and corresponding density distributions (Sch: schwannoma, Mets: metastasis, Men: meningioma, Epen: ependymoma). **e** Shows individual ROC curves for each tumor class. **f** Summarizes the ablation study (n = 36) comparing balanced accuracy of SpineXtract (self-supervision + transformer-based classification) against both the foundation model (self-supervision + linear classification) and state-of-the-art SRH-CNS classifier (supervision + linear classification), confirming SpineXtract’s superior performance across all tumor categories.

Demographic analysis revealed male predominance versus females, (median: 58 vs. 43 years). The cohort was primarily Caucasian, with East Asian and African American populations each constituting a small percentage. Institutional distribution of participants showed: MUV (19 individuals, 43.2%), UM (16 individuals, 36.4%), and NYU (9 individuals, 20.4%). (See Fig. 2 and Supplementary Fig. 2b-c).

### Histopathological findings and clinical parameters

Final pathological examination of the testing cohort identified a spectrum of spinal neoplasms with Spinal Ependymoma, CNS WHO Grade 2, and Myxopapillary Ependymoma, CNS WHO Grade 2 representing the predominant entities, followed by Schwannoma, CNS WHO Grade 1. Meningioma, CNS WHO Grade 1-2, constituted a portion of cases, while various metastatic lesions accounted for the remainder, originating from pulmonary, gastrointestinal, sarcomatous, and hematopoietic origins. Lesions were predominantly located in the intradural extramedullary compartment, with fewer instances in intramedullary and extradural locations. All participants underwent surgical excision as the initial therapeutic approach, with none receiving prior radiation treatment. Disease recurrence was documented in three individuals (see Supplementary Fig. 2c).

### Diagnostic performance of SpineXtract: International multicenter simulated single-arm clinical trial

From a total of 44 patients with 142 SRH slide-images examined, the test cohort comprised ependymoma (19 patients, 69 images, 48.6%), metastatic lesions (9 patients, 35 images, 24.6%), schwannoma (9 patients, 24 images, 16.9%), and meningioma (7 patients, 14 images, 9.9%).

At the aggregated patient-level, SpineXtract achieved a macro-averaged balanced accuracy of 92.9% (95% CI: 85.5–98.2) with a macro-AUROC of 98.0% (95% CI: 93.8–100.0). Sensitivity and specificity reached 89.4% (95% CI: 77.7–97.2) and 96.4% (95% CI: 90.9–100.0), respectively, confirming reliable cross-entity generalization. Prevalence-weighted estimates yielded a PPV of 87.5% (95% CI: 67.3–100.0) and NPV of 94.4% (95% CI: 89.3–98.4). To assess real-world applicability under minimal sampling conditions, a one-random-slide-per-patient analysis was performed. When restricted to a single SRH image per patient, SpineXtract maintained a balanced accuracy of 93.7% ± 1.2% (95% CI: 92.0–95.9).

Slide-level evaluation demonstrated a macro-averaged balanced accuracy of 92.4% (95% CI: 86.4–97.7) and AUROC of 96.5% (95% CI: 91.7–99.9). Sensitivity and specificity were 89.6% (95% CI: 81.3–96.6) and 95.2% (95% CI: 87.8–99.7), respectively.

### Entity-specific performance analysis exhibited variation across tumor types

Patient-level performance in meningioma and schwannoma achieved balanced accuracies of 98.6% (95% CI: 95.7–100) and 98.5% (95% CI: 95.3–100), respectively (sensitivity: 100%, specificity: 97.2% and sensitivity: 100%, specificity: 97.1%). Metastatic lesion identification showed a balanced accuracy of 90.5% (95% CI: 77.2–98.6%) (sensitivity: 89.5%, specificity: 91.5%), while ependymoma presented the greatest diagnostic challenge, achieving a balanced accuracy of 84.1% (95% CI: 73.6–94.4%) (sensitivity: 68.2%, specificity: 100%). The discriminative capabilities, as measured by AUROC, correlated with these performance patterns: schwannoma achieved perfect discrimination (100%), followed by meningioma (99.6%), metastasis (96.5%), and ependymoma (95.7%).

**Fig. 3:**
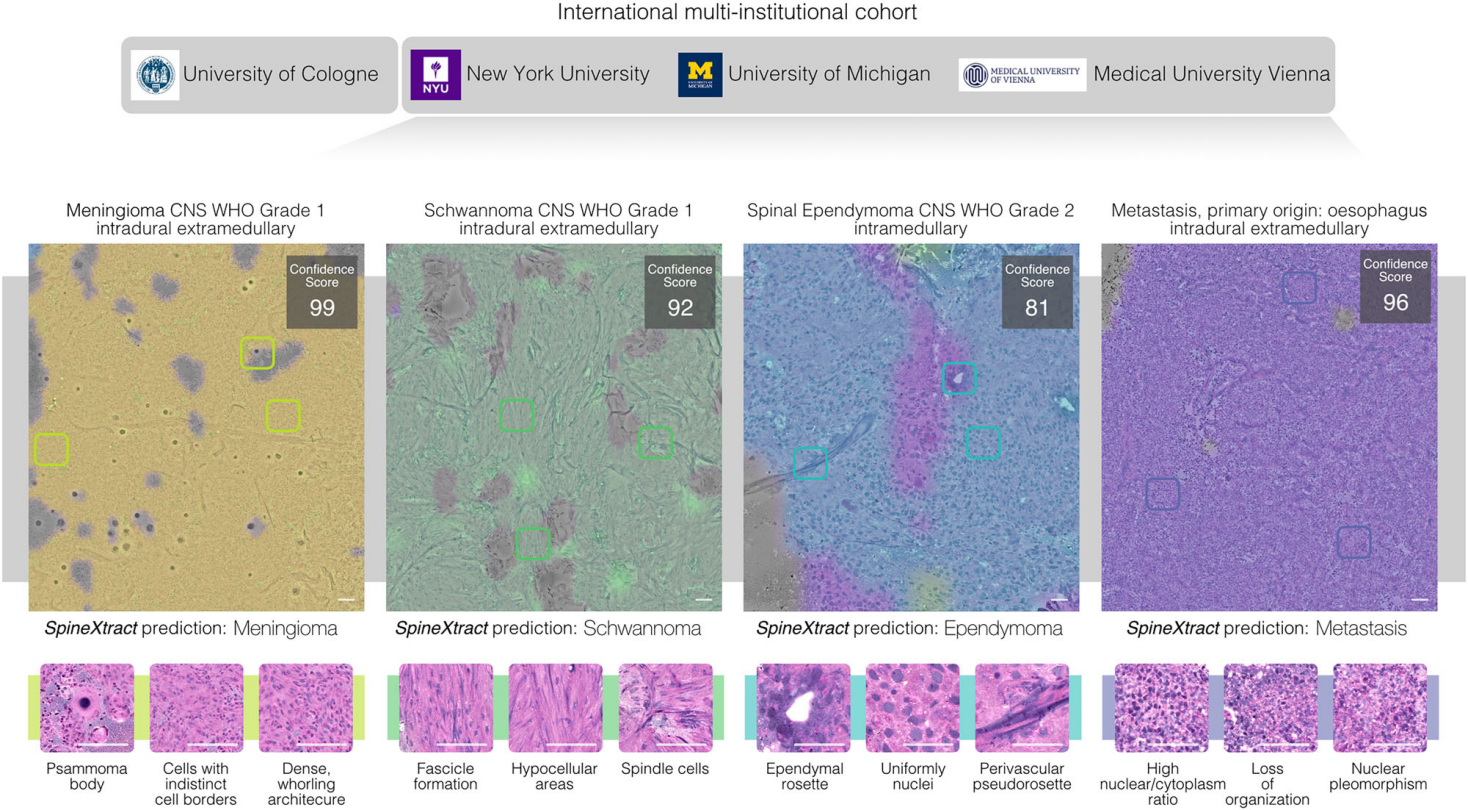
Interpretable AI-guided diagnosis of spine tumors across international institutions. Representative specimens from four common spine tumor types are shown with their corresponding confidence scores (maximum 100): meningioma (100), schwannoma (99), ependymoma (81), and metastasis (97). The model generates color-coded and interpretable visualization overlays that highlight diagnostically relevant regions specific to each tumor type. Below each prediction, characteristic histological features are displayed in magnified SRH patches, revealing distinctive patterns such as psammoma bodies in meningioma, fascicle formation in schwannoma, ependymal rosettes in ependymoma, and nuclear pleomorphism in metastasis. SpineXtract maintains consistent performance across institutions (New York, Michigan, Vienna), diverse anatomical locations (intradural extramedullary, intramedullary and extradural) and histological presentations, providing surgeons with immediate visual guidance during intraoperative decision-making.

At the slide level, corresponding balanced accuracies were 80.5% (ependymoma), 98.4% (meningioma), 91.1% (metastasis), and 99.1% (schwannoma), mirroring patient-level trends and confirming consistent discrimination across scales. (Figs. 2 and 3, Supplementary Fig. 3–4)

### Model discrimination, calibration, and clinical confidence thresholding

Calibration analysis showed well-calibrated probabilistic outputs, with an overall multiclass Brier score of 0.22 and class-wise patient-level values of 0.123 for ependymoma, 0.032 for meningioma, 0.058 for metastasis, and 0.025 for schwannoma. Corresponding expected calibration errors (0.147, 0.041, 0.073, and 0.041) indicated consistent reliability of predicted probabilities across tumor entities. Decision curve analysis demonstrated positive clinical net benefit over a broad range of threshold probabilities for all tumor classes, confirming the model’s utility for risk-stratified clinical decision-making across different diagnostic scenarios. Based on the confidence score distribution in the multicenter test cohort, an empirical threshold ($\tau$ = 0.77) was derived to guide future clinical implementation, enabling identification of low-confidence predictions. (Supplementary Fig. 5).

### Cross-institutional and demographic-stratified performance analysis

To assess institutional performance variability, we conducted a comprehensive subgroup analysis across our multicenter cohort. This evaluation examined the performance at each participating tertiary academic medical center, acknowledging the inherent variability in case distribution that characterizes real-world clinical practice. Our analysis revealed consistency in performance across institutions by patient-level macro-averaged balanced accuracy: MUV achieved 92.0% (95%CI: 83.3–100), followed by NYU at 91.6% (95%CI: 76.1–100) and UM at 91.4% (95%CI: 70.8–97.6). Institutional case distribution reflected: MUV (n = 19), NYU (n = 9), UM (n = 16). Note that NYU contained no meningioma cases and UM contained no schwannoma cases. Diagnostic performance metrics demonstrated consistent patterns with some institutional variation. Ependymoma detection showed moderate variation in sensitivity across centers (57.1% at MUV, 66.6% at NYU, 77.7% at UM), while maintaining excellent specificity (100% at all centers). For institutions with meningioma cases, perfect sensitivity (100%) was observed with strong specificity (92.8% at MUV, 100% at UM). Similarly, centers with schwannoma specimens achieved perfect sensitivity (100% at both MUV and NYU) with robust specificity (92.3% at MUV, 100% at NYU). Metastatic lesion identification showed excellent performance across all centers, with sensitivity ranging from 100% (MUV, NYU) to 80.0% (UM), alongside high specificity (94.4% at MUV, 83.3% at NYU, 90.9% at UM). Demographic stratification revealed consistent performance across patient subgroups, with balanced accuracy ranging from 91.4% in males to 93.5% in females, and from 90.5% to 93.4% across age groups (<30, 30-60, >60 years).

### Workflow timing

In a representative workflow evaluation, the complete process from specimen acquisition to AI-based prediction was completed within approximately five minutes. The individual steps required 30–60 seconds for tissue acquisition in the operating room and insertion into the laser imaging system, around three minutes for SRH image generation, and about one minute for AI inference and visualization (See Fig. 1).

### Prevalence sensitivity analysis

To examine model stability under varying epidemiological conditions, a prevalence-sensitivity analysis was conducted across three representative clinical prevalence scenarios: (a) the multicenter test cohort, dominated by ependymoma (45%) and schwannoma (30%); (b) general spinal oncology settings^16^, characterized by a predominance of metastases (60%); and (c) intradural-extramedullary-rich conditions^17^, augmented for schwannoma (45%) and meningioma (35%). Across scenarios, SpineXtract maintained consistent predictive performance. In metastasis-dominant settings, PPV for metastatic lesions increased by roughly 75% compared to the test cohort, while PPV for ependymoma declined by about 55% with a corresponding NPV gain of 25%. Under referral-clinic conditions, predictive values remained stable for schwannoma and meningioma, with PPV changes within ±10% and NPV consistently high. (Supplementary Fig. 6).

### Comparison between state-of-the-art SRH-CNS classifier versus SpineXtract for spinal neoplasm diagnosis

To establish a comparative performance baseline, we evaluated the state-of-the-art SRH-CNS classifier originally trained for intracranial CNS tumor classification against our specialized SpineXtract platform on a subset of 36 patients (slides=96) across the four spinal tumor types.

**Fig. 4:**
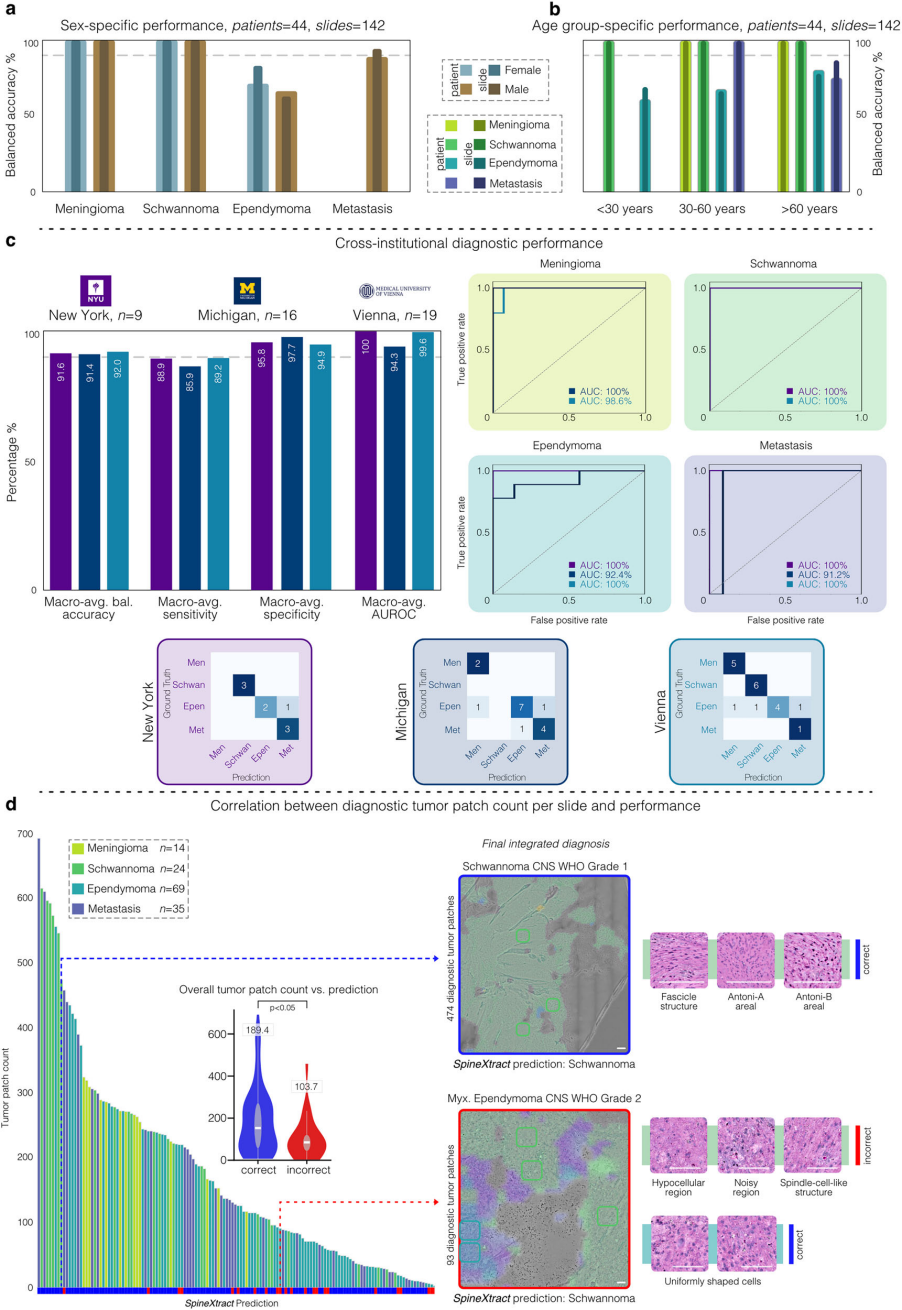
Performance benchmarking comparing SpineXtract against the state-of-the-art SRH CNS classifier. **a** Shows the spinal tumors test cohort composition (*n* = 36) used for patient-level comparison, with ependymoma being most prevalent, followed by metastasis, schwannoma, and meningioma. **b** Displays overall performance metrics across the entire test cohort (n = 36), demonstrating SpineXtract’s superior performance (blue bars) compared to the state-of-the-art SRH CNS classifier (gray bars) across all metrics, with particularly notable improvements in all macro-average metrics. **c** Provides detailed performance comparisons for each tumor type, showing SpineXtract consistently outperforming the general classifier across all four tumor categories, with particularly improvements for ependymoma sensitivity (+61.7%) and balanced accuracy (+30.8%), highlighting advantages of anatomical site-specific algorithms like SpineXtract for specialized clinical applications. **d** Confusion matrices demonstrate SpineXtract’s superior classification accuracy with fewer misclassifications compared to the state-of-the-art SRH CNS classifier, particularly reducing diagnostic errors and improving overall discrimination between spinal tumor types.

The performance comparison revealed substantial differences between the two approaches. SpineXtract demonstrated significantly superior performance with a mean balanced accuracy of 92.3% (95%CI: 87.4-94.2) compared to the SRH-CNS classifier’s 76.7% (95%CI: 47.5-100), representing an improvement of 15.6 percentage points. This performance differential was consistent across individual diagnostic classes and discriminative capabilities. Class-specific balanced accuracy improvements were most pronounced for ependymoma, where SpineXtract achieved 80.8% (95%CI: 68.5-85.9) compared to the SRH-CNS classifier’s 50.0% (95%CI: 47.2-55.8). For meningioma, both systems achieved excellent balanced accuracy, though SpineXtract showed superior performance (98.6%, 95%CI: 95.0-99.4 vs 81.0%, 95%CI: 72.5-92.3). Schwannoma classification demonstrated substantial improvement with SpineXtract reaching 98.2% balanced accuracy (95%CI: 96.6-100) versus 94.6% (95%CI: 71.6-92.7) for the SRH-CNS system. Metastatic lesion identification showed enhancement from 81.4% (95%CI: 66.1-86.2) to 92.0% (95%CI: 83.4-96.0) balanced accuracy. AUROC analysis revealed consistent discriminative superiority for SpineXtract across all tumor classes: meningioma (99.5% vs 100%), schwannoma (100% vs 100%), metastasis (95.9% vs 83.5%), and ependymoma (94.5% vs 64.1%), confirming the specialized model’s enhanced ability to distinguish between spinal tumor types. (Fig. 4 and Supplementary Fig. 7).

### Impact of tumor patch count on SpineXtract classification performance

**Fig. 5:**
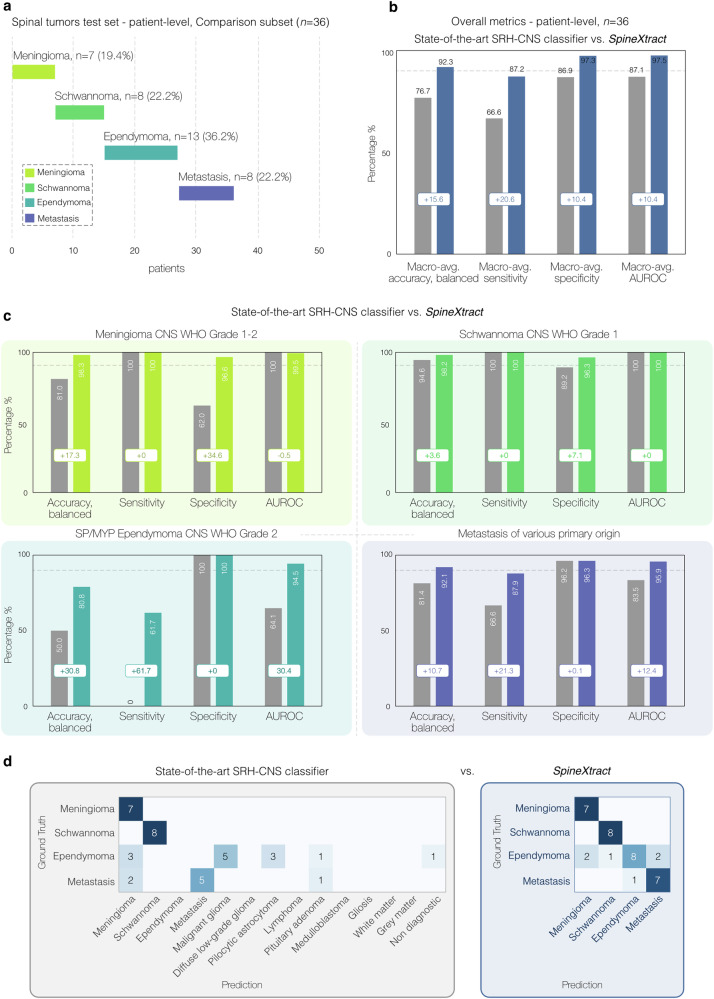
Demographic and institutional performance analysis of SpineXtract. **a** Sex-specific performance (*n* = 142) shows balanced accuracy across tumor types for both females and males, with slight variations in ependymoma classification. **b** Age group-specific performance reveals consistent accuracy across age cohorts (<30, 30-60, >60 years) for most tumor types. **c** Cross-institutional diagnostic performance compares metrics across three tertiary medical centers (New York, Michigan, Vienna) demonstrating robust generalization with strong ROC curves for all tumor types at each institution and corresponding confusion matrices. **d** Correlation between tumor patch count and diagnostic performance reveals higher patch counts generally yield more accurate predictions (189.4 patches for correct vs. 103.7 for incorrect predictions, *p* < 0.05), with contrasting heatmap examples of diagnostic integration shown for correctly identified schwannoma and incorrectly classified myxoid ependymoma, highlighting the importance of diagnostic feature recognition in challenging cases.

To investigate factors influencing model performance, we analyzed the relationship between diagnostic tumor patch count and prediction accuracy. Mean patch count for correct predictions (189.4) was significantly higher than for incorrect predictions (103.7; *p* = 0.00948). Logistic regression confirmed that each additional patch increased correct prediction probability by 0.533% (OR = 1.005, 95%CI: 1.001-1.009, *p* = 0.01858). Accuracy improved with higher patch counts, ranging from 64.0% (50-99 patches) to 95.2% (300+ patches). Entity-specific analysis revealed this relationship was most pronounced for ependymoma, where mean patch count differed significantly between correct (152.1) and incorrect (85.5) predictions, suggesting that larger tissue samples enhance diagnostic confidence for this tumor type (Fig. 5d).

## Discussion

We developed SpineXtract, the first SRH-based classifier designed explicitly for spinal tumors, which differentiates the four most common types of spinal tumors using Raman spectromics. Using a self-supervision learning strategy combined with a modified self-attention module for patch-based classification, we demonstrated not only SpineXtract’s superiority to the state-of-the-art SRH-CNS tumor classifier but also proved the modified self-attention module within our pipeline as essential part in achieving superior mean balanced accuracy. Additionally, for enhanced clinical usability and model interpretability, results are visualized through color-coded heatmaps and combined with a confidence score providing intraoperative visual feedback and enabling rapid decision-making during spinal tumor surgery for surgeons and pathologists.

The clinical relevance of SpineXtract is particularly significant in the context of complex spinal cases, rather than standard presentations (See Supplementary Fig. 8). Despite advances in imaging techniques, spinal tumors are frequently misclassified due to atypical radiographic features, underscoring that definitive diagnosis relies exclusively on histopathological examination. Current intraoperative diagnostic methods like frozen section analysis are time-intensive exceeding 30 minutes and more on average, depend on neuropathologist availability, undergo subjective review and are limited to regular working hours^4,5^. SpineXtract provides surgeons with a diagnosis within 5 minutes (turnaround time may vary across institutions depending on operator experience and slide-image size), while maintaining superior diagnostic accuracy with over 92% compared to conventional frozen section analysis (86.6-88.6% overall) reported in the literature^5–7^. Even when restricted to a single randomly selected SRH slide per patient, diagnostic accuracy remained high, emphasizing the model’s robustness to sampling variability. Importantly, all reported performance metrics were derived from calibrated probability outputs, ensuring clinically meaningful and interpretable predictions. Calibration and decision-analytic evaluations confirmed reliable probability estimates and consistent net benefit across a broad range of thresholds. Together with the prevalence-sensitivity analysis, which showed stable predictive behavior under both metastasis-dominant and intradural-extramedullary-rich conditions, these results underscore the generalizability and clinical reliability of SpineXtract across diverse real-world settings. Furthermore, while the evolving AI-based Raman spectromics has proven successful for intracranial CNS tumors, spinal tumors have remained largely unaddressed^10,11,14^. We here demonstrate, for the first time, that the pre-existing SRH-CNS tumor classifier cannot be reliably applied to corresponding CNS tumors in the spinal axis. SpineXtract addressed this anatomical site-specific gap by enabling rapid and accurate intraoperative analysis, providing time-efficient diagnosis during surgery.

To further validate SpineXtract we evaluated each tumor entity using a multicentric testing cohort and compared its performance to the state-of-the-art SRH-CNS tumor classifier. In general, meningiomas account for approximately 25% of spinal tumors^18^. Both SRH-CNS classifier and SpineXtract showed high sensitivity, likely due to similar histopathological features in intracranial and spinal meningioma. SpineXtract achieved higher specificity, likely because the SRH-CNS tumor classifier frequently misclassified other entities as meningioma. Spinal schwannoma are benign nerve sheath tumors that originate from Schwann cells and exhibit histopathological variants, for example cellular schwannomas mimicking malignant neoplasms^19^. Currently, there are no notable histomorphological differences between cranial and spinal tumors reported in current literature other than a higher frequency of the cellular subtype in the spinal region^20^. However, the SRH-CNS classifier was found to frequently misclassify spinal schwannoma, whereas SpineXtract was found to reach near perfect sensitivity and a balanced accuracy of over 98%. In contrast, spinal ependymoma arises from ependymal cells and appears extra- and intramedullary throughout the spinal axis, generating wide histomorphological heterogeneity across subtypes. Correspondingly, SpineXtract was most challenged in subsuming this heterogeneous morphology, particularly in intramedullary located lesions, achieving a balanced accuracy over 80.8% —still superior to the SRH-CNS classifier, which misclassified all spinal ependymomas. This discrepancy may reflect morphological overlap with other tumor types, e.g., resembling schwannomas via spindle cell-like/fascicular architecture (Supplementary Fig. 4)^21^. Following liver and lung the spine is the third most common site of metastasis formation, the most common among osseous metastasis locations, frequently originating from lung, breast, and prostate cancer as well as urinary system and hematopoietic origin - though reported distributions vary^22^. We found that the current SRH-CNS classifier achieved a limited sensitivity of only 81.4% versus SpineXtract with over 92% in predicting metastasis within our spinal tumor cohort. This reduced performance may be attributed to the limited diversity of metastatic tumor types in the SRH-CNS classifier’s training dataset, which was primarily focused on intracranial lesions^10^. While metastatic histomorphology remains mostly consistent regardless of anatomical location, the spectrum of primary malignancies metastasizing to spinal versus intracranial sites differs. SpineXtract was trained on a broader representation of secondary malignancies, including those from prostate and hematopoietic primaries, despite their absence in the current test cohort. Notably, even metastases from non-small cell lung cancer—which commonly spread to both intracranial and spinal sites—were often misclassified by the SRH-CNS classifier in our cohort due to site-specific microenvironmental pressures potentially leading to variations in histomorphological appearance, thus requiring anatomical site-specific training for optimal recognition^23^.

SpineXtract even correctly categorizes rare spinal tumor subtypes unseen during training, including clear cell meningioma and metastasis of an ewing sarcoma. This demonstrates zero-shot generalization and particular clinical relevance, as our cohort contained ambiguous cases where preoperative imaging and intraoperative macroscopic observation yielded completely different suspect diagnoses. In such challenging scenarios, SpineXtract provides microscopic certainty for accurate decision-making (Supplementary Fig. 8).

Furthermore, our analysis revealed a significant correlation between diagnostic tumor patch count and classification accuracy (p = 0.00948), with correctly classified cases utilizing 82.7% more patches than misclassified cases (189.4 vs 103.7). Each additional patch increased correct prediction odds by 0.533% (OR = 1.00533, p = 0.01858), particularly in ependymomas, reflecting biological heterogeneity where certain categories require more extensive sampling, with both patch quantity and quality influencing performance^15^.

We focused on the four most common spinal tumor entities due to limited case availability, thereby excluding rarer differentials such as spinal astrocytomas or lymphomas, which represent promising directions for future research. SpineXtract was developed and validated using SRH slide-images; however, the framework is readily adaptable to digitized H&E and immunohistochemistry data, supporting future multimodal integration. The test cohort exhibited an ependymoma-predominant distribution rather than the typical epidemiological pattern of spinal tumors (metastasis > meningioma > schwannoma > ependymoma), reflecting the case mix of tertiary neurosurgical centers that frequently manage diagnostically complex cases. Importantly, the prevalence-sensitivity analysis demonstrated stable predictive performance across diverse epidemiological scenarios, including metastasis-dominant conditions. While the comparison with conventional frozen section analysis was based on reported benchmarks rather than a direct head-to-head study, these results establish a strong reference point for subsequent prospective intraoperative comparisons.

To ensure safe and reliable intraoperative deployment, SpineXtract integrates a calibrated empirical confidence threshold (τ = 0.77) derived from the multicentric test cohort. Predictions below this threshold automatically trigger intraoperative feedback for repeat imaging, additional sampling, or expert review, preventing low-confidence classifications from influencing autonomous decision-making. This built-in safeguard, combined with calibrated probability outputs, ensures interpretability, maintains patient safety, and aligns with current principles for responsible clinical AI implementation. Finally, external validation in routine community settings with more representative prevalence patterns will be essential to confirm the generalizability and clinical scalability of SpineXtract.

SpineXtract achieves high accuracy for rapid intraoperative spinal tumor diagnosis across institutions, outperforming the existing SRH-CNS tumor classifier. As the first spinal classifier for digital histopathology using a modified self-attention mechanism, it handles histomorphological heterogeneity within tumor entities. SpineXtract integrates seamlessly into surgical workflows, providing reliable predictions within five minutes in total to enhance diagnostic and treatment efficiency.

## Methods

### Study design

Following TRIPOD + AI and STARD-AI guidelines^24,25^ this study aimed to (1) create SpineXtract, an advanced intraoperative diagnostic system utilizing stimulated Raman histology (SRH) with a fully automated AI workflow for spinal tumor identification; (2) conduct an international multicenter simulated single-arm clinical trial using existing SRH data to assess overall, entity-specific, and demographic-stratified diagnostic performance; and (3) evaluate this tool against existing AI-based SRH-CNS classification framework. SRH research was IRB-approved across all centers (see ethics statement) with patient recruitment beginning June 2015 until January 2025. Inclusion criteria comprised: (1) suspected spinal (non)-neoplastic tissue from University of Cologne (UKK), New York University (NYU), University of Michigan (UM), and Medical University of Vienna (MUV); (2) informed consent; and (3) additional specimen beyond standard diagnostic requirements. We developed, validated, and evaluated our model on SRH slide-image scans to deliver fast intraoperative diagnoses of fresh, unprocessed, spinal tissue specimens via a portable label-free SRH imaging device available at each institution site. We also implemented semantic segmentation for real-time visual interpretation of SRH slide-image predictions for surgeon and pathologist review. Neither patients nor funding sources/sponsors had any influence on study design, data collection, data analysis, data interpretation, or writing of the report. The study was approved by the Ethics Committees of all participating centers: the Ethics Committee of NYU Langone Health (s19-01931); the Ethics Committee of the University of Michigan (HUM00083059, HUM00005731); the Ethics Committee of the Medical University of Vienna (419/2008); and the Ethics Committee of the Faculty of Medicine, University of Cologne (21-1238).

### Tissue processing, Raman spectromics and stimulated Raman histology

The whole-slide images for AI pipeline development and the simulated multicenter clinical trial were acquired using a portable fiber-laser-based stimulated Raman scattering microscope (NIO Laser Imaging System, Invenio Imaging Inc., Santa Clara, CA, USA) at each institution for intraoperative visualization of Raman spectromics^8,9^. This advanced system employed a dual-wavelength fiber laser configuration with a fixed 790 nm pump beam and a precisely tunable Stokes beam (1015-1050 nm), enabling comprehensive coverage of Raman shifts between 2800-3130 cm-1 for detailed molecular analysis. For optimal imaging conditions, fresh, unprocessed, label-free tissue specimens were carefully mounted on acrylic slides and gently compressed with cover slips to ensure consistent sample thickness. High-resolution imaging was performed using sophisticated beam scanning technology at 450 nm pixel-1 resolution, maintaining a 1000-pixel width per imaging strip at an acquisition speed of 0.4 Mpixel/second/Raman shift. The imaging protocol strategically utilized two specific Raman shifts that were sequentially captured: 2845 cm-1, which generates strong SRS contrast from CH2 symmetric stretching vibrations in fatty acids (providing detailed visualization of lipid-rich regions, designated as the CH2 channel), and 2930 cm-1, which highlights cellular CH3-rich areas (specifically enhancing DNA and protein-rich regions, designated as the CH3 channel). These raw grayscale SRS images underwent algorithmic transformation into virtual H&E-like stimulated Raman histology (SRH) images for enhanced visualization on the portable system’s display interface, following previously established methodologies (See Supplementary Fig. 1a).

The image preprocessing pipeline began with the high-precision 16-bit grayscale CH2 channel images, which underwent systematic pixel-by-pixel subtraction from the corresponding grayscale CH3 channel images. This mathematical operation was followed by strategic concatenation to produce a comprehensive three-channel composite image (consisting of 2930 cm-1 minus 2845 cm^-1^; 2845 cm^-1^; and 2930 cm^-1^), which enhanced molecular contrast while preserving all diagnostic tissue information. For deep learning algorithm development, we implemented a sophisticated 300×300-pixel sliding window approach with a consistent 300-pixel stride in both horizontal and vertical directions. This methodical approach generated uniform, non-overlapping 300 × 300-pixel image patches that precisely matched the required input dimensions for the vision encoder architecture, ensuring optimal data formatting during both the training and inference phases of the artificial intelligence system.

### Image training dataset

Training used preprocessed image patches from a single SRH imager at UKK. For model development, we utilized 1258 SRH slide-images spanning the entire neuro-oncological spectrum with at least 15 entities. For fine-tuning, we filtered common spinal tumor types: (a) Meningioma (CNS WHO grade 1-2, n = 58), (b) Schwannoma (CNS WHO grade 1-2, including Hybrid nerve sheath tumor, n = 53), (c) Ependymoma (including myxopapillary and spinal, CNS WHO grade 2, n = 40), and (d) Metastatic tumors (from lung, kidney, bladder/urinary tract, skin, colorectal, esophagus, prostate, breast and hematopoietic system, n = 47), resulting in 198 SRH slide-images for downstream training. There was no overlap of patients or slides between the training/fine-tuning data and the external multicenter test cohort. A previously trained segmentation algorithm categorized patches as tumor tissue, normal brain tissue, or non-diagnostic regions^11^. We accepted images containing up to 90% non-diagnostic patches to reflect real-world variations while ensuring at least 10% diagnostically relevant tissue per image for training. Images lacking diagnostic patches or containing more than 50% normal healthy tissue were excluded. (See Supplementary Fig. 2a-b).

### International multicenter simulated clinical trial design

We evaluated SpineXtract’s performance through an international multicenter simulated single-arm clinical trial with macro-average balanced accuracy $(\,\frac{1}{4}{\sum }_{i=1}^{4}\frac{{Sensitivity}+{Specificity}}{2})$ as the primary endpoint performed at patient-level. Secondary endpoints included slide-level performance metrics and post-hoc stratified analyses by tumor entity, institution, demographic variables (sex and age), and benchmarking against previous AI-based SRH-CNS classification system^11^ (available on each site’s clinical SRH imager, with 14-class prediction capacity), comparing our transformer-based classification versus linear approach, and assessing the model’s ability to differentiate (a) meningioma, (b) schwannoma, (c) ependymoma, and (d) metastasis. (See Supplementary Materials p 2). In this simulated trial approach, the trained SpineXtract model/code was shared across centers for local application to their respective existing SRH image datasets, allowing patient data and images to remain within each respective institution. External testing data came from NYU, UM, and MUV, with collection ending January 2025. Inclusion criteria comprised: (1) adults 18+ with suspected primary or secondary (non)-neoplastic spinal lesion (extra- or intramedullary) based on preoperative imaging, specifically those suspected of the four categories; (2) patients scheduled for surgical intervention; (3) ability to provide informed consent. Exclusion criteria: (1) inability to provide consent to SRH research; (2) slide and tissue damage during preparation; (3) only non-diagnostic areas in SRH images. Sample size determination was based on preliminary testing of the existing SRH-CNS classifier on spinal data (n = 265 slide-images, UKK) which showed a mean balanced accuracy of 72.8%. Power analysis indicated that at least 142 slide-images in total would provide 95% power to detect an improvement to 90% mean balanced accuracy with our new model (α = 0.05). All diagnoses were derived from standard formalin-fixed, paraffin-embedded tissue distinct from the SRH specimen used for AI inference. In cases of ambiguous or mosaic histology, consensus was reached via multi-reader review and, if necessary, additional molecular testing following WHO 2021 criteria. No SRH data informed the ground-truth decision process. Preprocessing and augmentations were performed identically across all institutions and devices, ensuring site-agnostic image handling.

### Deep learning pipeline and development

We employed a broadly applicable and comprehensive visual learning approach that utilized a diverse SRH image foundation representing nearly the complete neuro-oncological spectrum available at UKK institution. (See Supplementary Fig. 2a). Current research supports this approach for establishing a robust foundation for visual patch-level representation learning and subsequent downstream classification applications. In selecting our foundation model architecture, we chose ResNet50 based on our previous empirical findings demonstrating that BYOL-based self-supervision with ResNet50 consistently outperformed Vision Transformers when fine-tuning on limited annotated datasets with few classes. While Vision Transformers offer superior computational efficiency for large-scale datasets, our experiments revealed that ResNet50’s architectural inductive biases are particularly well-suited for medical imaging scenarios where labeled data is scarce and the number of target classes is small^15^. For our learning methodology, we implemented an advanced self-supervised approach similar to the BYOL (Bootstrap Your Own Latent) framework^26^. The principal advantage of this method is its elimination of the requirement for negative pairs, making it appropriate for histopathological datasets, in contrast to alternative contrastive learning methods like SimCLR and SupCon^27^. This learning system was composed of two essential integrated elements: a student network and a teacher network.

The network asymmetry manifests in the network structure where the student pathway contains three components: an encoder network ${f}_{\theta }$, a projector network ${g}_{\theta }$, and a predictor network ${q}_{\theta }$. In contrast, the teacher pathway contains only two components: an encoder network ${f}_{\xi \,}$ and a projector network ${g}_{\xi }$, deliberately omitting the predictor component. (See Supplementary Fig. 1b).

This asymmetric design is fundamental to preventing representation collapse. For each patch image we sample two independent stochastic augmented views. Each view is drawn independently from the following pool (probalities/parameters in Supplemental Training Protocol): random horizontal/vertical flip, random sharpness, Gaussian blur, Gaussian noise, random autocontrast, random solarize, random erasing, random affine, and random resized crop. The student branch processes augmented view ${x}_{1}$ through its complete pipeline with encoding, projection and prediction: $y={f}_{\theta }\left({x}_{1}\right),z={g}_{\theta }\left(y\right),p={q}_{\theta }\left(z\right)$. Meanwhile, the teacher branch processes view ${x}_{2}$ but stops at projection: ${y}^{{\prime} }={f}_{\xi }\left({x}_{2}\right),{z}^{{\prime} }={g}_{\xi }\left({y}^{{\prime} }\right)$. The similarity loss is defined as the normalized negative cosine similarity, following the original BYOL formulation^26^, and compares the student’s prediction with the teacher’s projection: ${{\mathcal{L}}}_{\theta ,\xi }=2-2\cdot \frac{\left(p,{z}^{{\prime} }\right)}{\parallel p{\parallel }_{2}\,\cdot \,\parallel {z}^{{\prime} }{\parallel }_{2}}$. This forces the student’s predictor to align with the teacher’s projection space, creating a challenging learning task that drives invariant SRH visual representation learning without explicit labels.

The optimization employs a key asymmetry: student parameters $\theta$ update via direct gradient descent with $\theta \leftarrow {optimizer}\left(\theta ,{\nabla }_{\theta },{{\mathscr{L}}}_{\theta ,\xi },\eta \right)$, while teacher parameters $\xi$ update only through exponential moving average $\xi \leftarrow \tau \xi +\left(1-\tau \right)\theta$ without receiving gradients. This creates a slowly evolving target representation that stabilizes training and prevents collapse. After training, only the encoder ${f}_{\theta }$ is forwarded for downstream tasks, discarding both the projector and predictor networks.

Prior to the training process, a smaller dataset comprising 10% of the entire UKK data pool was created as a validation dataset to monitor training and performance. Various hyperparameter modifications, such as optimizer and learning rate scheduler, were applied to enable optimal visual representation foundation model training and training was stopped after 700 epochs. (See Supplemental Training Protocol).

### Transformer-enhanced multiclass classification

To leverage our SRH foundation model, we freezed its weights and utilized the resulting patch embeddings as input for a transformer-based classification approach^28^. Each e.g., $k-{th}$-dimensional embedding is restructured into sequential form to capture complex relationships within each patch for final classification into the most common four spinal tumor categories: (1) meningioma, (2) schwannoma, (3) ependymoma, and (4) metastasis (see Supplementary Fig. 2b).

We modified this self-attention mechanism by consuming each $k-{th}$-dimensional patch embedding specifically to address a crucial limitation of conventional linear classification approaches. By treating segments of the embedding vector as tokens in a sequence, the self-attention mechanism learns to focus on the most diagnostically relevant portions of the embedding while de-emphasizing less informative regions. We hypothesized that this selective attention capability is particularly valuable for our histopathological task, where subtle tissue patterns may occupy only a fraction of the embedding space but carry significant diagnostic value. The attention mechanism computes ${Attention}\left(Q,K,V\right)={softmax}\left(\frac{{{QK}}^{T}}{\sqrt{{d}_{k}}}\right)V$ across multiple projection subspaces^28^. This allows the model to simultaneously attend to different feature subspaces, effectively learning which components of the $k-{th}$ -dimensional embedding vector contain discriminative information for distinguishing between the four spinal tumor types. For instance, certain regions of the embedding may capture nuclear morphology relevant for meningioma identification, while others may represent architectural patterns critical for distinguishing metastases. (See Supplementary Fig. 1c).

The transformer architecture effectively transforms the raw embeddings into a more classification-appropriate representation by reweighting features according to their relevance for the downstream task. This transformation provides the classification MLP with an optimized feature space where discriminative tumor characteristics are emphasized. The classification multi-layer perceptron ${MLP}$ uses a simple single-layer network:$\,{P}_{{Meningioma},{Schwannoma},{Ependymoma},{Metastasis}}={ML}{P}_{{classes}}={softmax}\left(W* {transformer}\left(x\right)+b\right)$ where softmax directly maps to tumor class probabilities. We fine-tuned the model using a cross-entropy loss between the true histopathological labels—established from H&E, immunohistochemistry (IHC), and molecular diagnostics on formalin-fixed paraffin-embedded (FFPE) sister tissue specimens of the same tumor—and the predicted class probabilities. Neuropathologists were blinded to SRH and AI outputs, and SRH information was not used to derive or confirm histopathological labels in any training case: ${{\mathscr{L}}}_{(y,P)}=-{y}_{{Meningioma}}* \log ({P}_{{Meningioma}})-{y}_{{Schwannoma}}* \log ({P}_{{Schwannoma}})-{y}_{{Ependymoma}}* \log ({P}_{{Ependymoma}})-{y}_{{Metastasis}}* \log ({P}_{{Metastasis}})$.

Various hyperparameter modifications, such as optimizer and learning rate scheduler, were applied to enable optimal fine-tuning of our SRH foundation model. During model development, validation set performance (10% of training data) showed convergence at epoch 10 with patch-based validation mean class accuracy of 83.3% and was selected as final model. (See Supplementary training protocol).

### Semantic segmentation and visual feedback generation

We developed a comprehensive visualization system to enhance intraoperative visual feedback and decision-making during spinal tumor surgery. Using previous visualization methods, our approach combines semantic segmentation in a clinically applicable workflow. Our visualization system employs a non-overlapping window technique across SRH images for multi-class semantic segmentation. For each position in the image, we extract a fixed-size patch of 300 pixels to be analyzed by SpineXtract model. The window function is defined as ${W}_{i,j}=I\left[i:i+{patch},j:j+{patch}\right]$ moving with a step size of 300 pixels across both dimensions to ensure no overlap between adjacent patches. For probability heatmap generation, each extracted patch is processed through our model to generate probabilities for each class: ${probaility}(c|{W}_{i,j})=\frac{{e}^{{z}_{c}}}{{\sum }_{c=1}^{C}{e}^{{z}_{c}}}$ where ${z}_{c}$ is the logit output for class $c$, and $C=4$ representing the total number of classes (meningioma, schwannoma, ependymoma, metastasis). We initialize an empty heatmap array $H\in {{\mathbb{R}}}^{{heightxwidthxC}}$ of the same size as the SRH image with an additional dimension for each class and populate it with patch probabilities. For color-coded visualization, we assign a distinct color map to each class: ${C}_{{map}}={c}_{{Meningioma}}\to {{color}}_{1},{c}_{{Schwannoma}}\to {{color}}_{2},{c}_{{Ependymoma}}\to {{color}}_{3},{c}_{{Metastasis}}\to {{color}}_{4}$. The final visualization overlay is created using a weighted sum: $V=\left(1-\alpha \right)\cdot I+\alpha \cdot {\sum }_{c=1}^{C}H\left[:,:,c\right]\cdot {C}_{{map}}\left(c\right)$, where ${\rm{\alpha }}$ represents an adjustable transparency factor. For whole-image classification, we aggregate patch-level probabilities: $P(c{|}I)=\frac{1}{N}{\sum }_{i,j}P{(}c{|}{W}_{i{,}j}{)}$, where $N$ is the total number of patches analyzed and patch coloring is based on the class with highest softmax proballity.

Beyond heatmap visualization, a per-slide confidence score is computed as the maximum calibrated class probability ${P}_{\max }=\,{{\max }_{i}P}_{i}$ after Platt scaling, reflecting the model’s most likely class prediction. For threshold calibration, slide-level confidences were aggregated to patient-level using patch-count-weighted means: ${C}_{{patient}}=\,\frac{{\sum }_{s}{n}_{s}{C}_{s}}{{\sum }_{s}{n}_{s}}$, where ${n}_{s}$ is the number of patches per slide. To establish an empirical threshold for low-confidence predictions, the patch-count-weighted mean confidence was calculated separately for correctly and incorrectly classified cases in the test cohort with $\tau =\frac{{C}_{{correct}}+{C}_{{incorrect}}}{2}$. Predictions with ${C}_{{patient}} < \tau$ are flagged for additional tissue sampling or repeat imaging.

This approach provides detailed spatial information through heatmap visualization and an interpretable confidence metric, enabling intuitive and reliable interpretation of SpineXtract predictions during spinal tumor surgery while maintaining computational efficiency for near real-time application (see Supplementary Fig. 1d).

### Statistics and reproducibility

We performed a two-sided power analysis for the multicenter simulated single-arm trial using a web-based calculator (https://imsiewebarchiv.uni-koeln.de/beratung/rechner/ps.html). To evaluate SpineXtract’s diagnostic performance against the final histopathological diagnosis (ground truth), we computed multiclass (macro-average) and per-class balanced accuracy ($\frac{{Sensitivity}+{Specificity}}{2})$, sensitivity, specificity, positive and negative predictive values, and AUROC with corresponding standard deviations and 95% level- and cluster-robust bootstrap (n = 1000 iterations) confidence intervals. Patch-level logits were transformed into softmax probabilities and summed to obtain slide- and patient-level probability vectors. Slide- and patient-level mappings were established using unique patient identifiers for hierarchical aggregation. Model calibration was performed via Platt scaling^29^ fitted on the spinal training cohort. Multiclass metrics were derived from calibrated argmax outputs, whereas per-class metrics and decision curve analysis (DCA) were based on calibrated probabilities. A per-class operating threshold of 0.5 was defined after calibration, ensuring reproducibility and calibration-consistence for deployment. Calibration reliability was further assessed using per-class calibration plots and Brier scores to quantify the agreement between predicted and observed probabilities. To control for oversampling bias, a one-random-slide-per-patient analysis was performed. The model confidence score reflected the top calibrated probability per SRH slide-image. To assess robustness under varying epidemiological conditions, prevalence-sensitivity analyses simulated PPV/NPV across different prevalence scenarios. We visualized learned feature representations using t-SNE dimensionality reduction and analyzed the effect of diagnostic patch count on classification accuracy using the Mann–Whitney U test and logistic regression (p < 0.05 considered significant).

## Supplementary information

Supplementary information

Supplementary information

## Data Availability

Because the raw SRH image datasets contain identifiable information within the original image files, they cannot be shared publicly. However, de-identified SRH patch images and corresponding diagnostic labels are available from the corresponding author upon reasonable request. Data access requires a data-use agreement between the requesting institution and the respective contributing centers (UKK, NYU, UM, and MUV) to ensure compliance with institutional and data-protection standards.

## Code availability

The complete SpineXtract training, inference, and evaluation pipeline, including model weights, is publicly available on GitHub (https://github.com/DavidReineckeMD/SpineXtract).
