# Supplementary material for: AI-driven label-free Raman spectromics for intraoperative spinal tumor assessment
Source: NPJ Digit Med. 2026 Mar 17;9:227. doi: 10.1038/s41746-025-02279-6 (PMC12996391; doi:10.1038/s41746-025-02279-6)
Supplement: Supplementary file 1 — Supplementary information [file 41746_2025_2279_MOESM1_ESM.pdf]

Supplementary Material

Table of Content

SUPPLEMENTARY MATERIALS 2

1. STARD Flow Diagram **2**

2. Computational hardware and software **2**

3. Supplemental training protocol **3**

Base Configuration 3
Optimizer Settings 3
Scheduler and Model-specific Parameters 3
Architecture Specifications 3
Training Details 4
Common Training Augmentations 4
Calibration and Deployment Parameters 5

SUPPLEMENTARY FIGURES 6

Supplementary Materials

1. STARD Flow Diagram

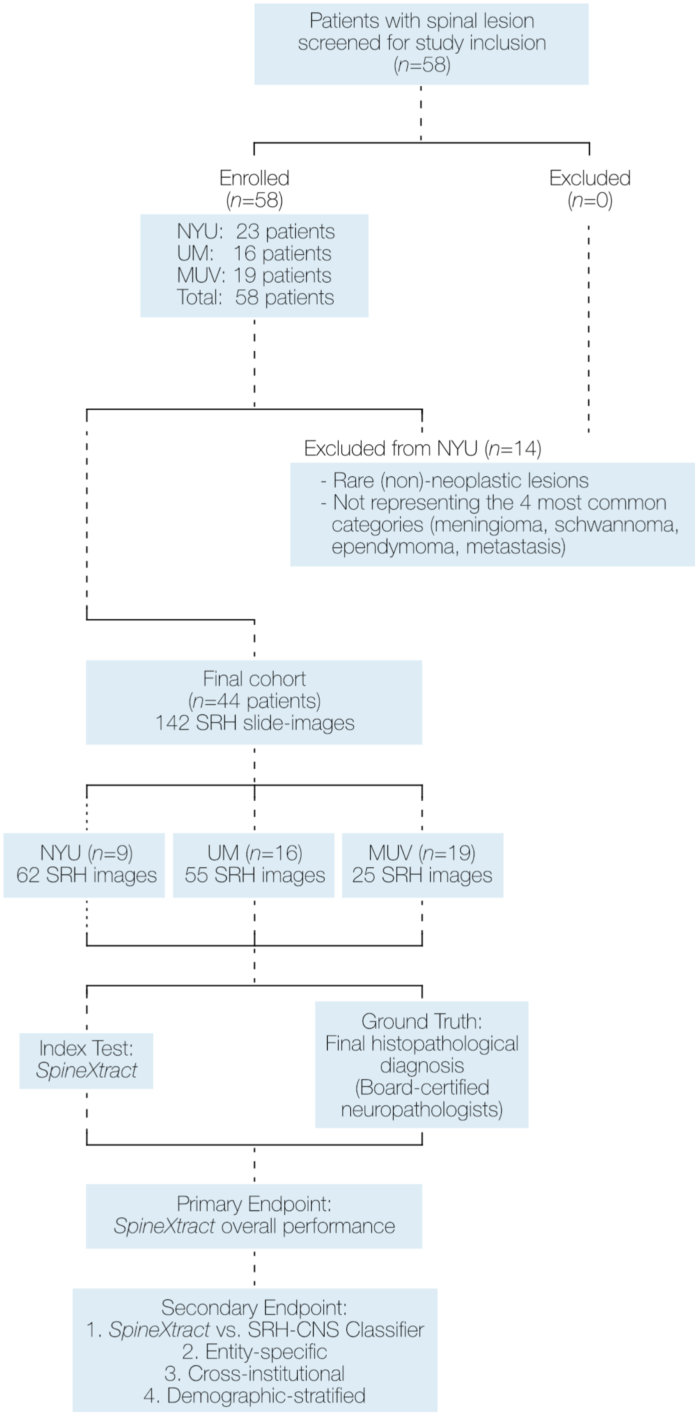

2. Computational hardware and software

Processing of all training and validation patch images was performed on a single Nvidia A100 80GB graphical processing unit with 4 x 16GB central processing units using our custom-developed SpineXtract package based on Python (version 3.10.13). To process SRH images from the NIO Laser Imaging System, we utilized the Pydicom package (version 2.4.3). All processed images were archived as 16-bit TIFF files on site-specific high-performance computing clusters for subsequent analysis using the tifffile package (version 2023.9.26). Our AI models were trained on four Nvidia A100 80GB GPUs on a high-performance cluster that complies with privacy regulations. The model architecture was implemented in PyTorch (version 2.1.2), using a customized untrained ResNet-50 model from

torchvision (0.10.10). Inference was performed on-site using an identical containerized environment (including CUDA/cuDNN and PyTorch versions), ensuring reproducibility across all centers. Performance metrics for model predictions during training and inference were calculated using Scikit-learn (version 1.4.0). Model inference was successfully tested on GPU and CPU architectures, demonstrating computational flexibility for different deployment scenarios. Complete details about additional dependencies, model weights, and technical specifications can be found in our open-source GitHub repository (https://github.com/DavidReineckeMD/SpineXtract).

3. **Supplemental training protocol**

Base Configuration

Parameter	BYOL (Self-supervised)	Transformer-MLP (Classification)
Seed	1000	1000
Input size	300 × 300 px	300 × 300 px
Batch size	256	256
Epochs	700	10
Training precision	bf16-mixed	bf16-mixed
Deterministic	False	False

Optimizer Settings

Parameter	BYOL (Self-supervised)	Transformer-MLP (Classification)
Optimizer type	AdamW	AdamW
Learning rate	0.001	0.001
Betas	[0.9, 0.999]	[0.9, 0.999]
Weight decay	0.07	0.07
Epsilon	1e-8	1e-8
Gradient clipping (norm)	1.0	1.0

Scheduler and Model-specific Parameters

Parameter	BYOL (Self-supervised)	Transformer-MLP (Classification)
Scheduler type	Cosine annealing with warm-up	Cosine annealing with warm-up
Warm-up steps	10 %	5 %
Cycles	0.5	0.5
Objective	Similarity loss	Cross-Entropy loss
EMA β	0.92	N/A
Prediction dimension	2048	4 (classes)
Early stopping	N/A	Patience = 5 epochs (validation loss plateau after epoch 5; best epoch = 10)

Architecture Specifications

Parameter	ResNet-50 (Backbone)	Transformer-MLP (Classification Head)
Type	ResNet-50 (custom, untrained)	Transformer-MLP
Embedding dimension (n_in)	2048	2048
Tokenization	N/A	Embeddings split into 32-D tokens (64 tokens per embedding; 2048 ÷ 32 = 64 for transformer self-attention module)

Parameter	ResNet-50 (Backbone)	Transformer-MLP (Classification Head)
Sequence length	N/A	64
d_model (token dimension)	N/A	32
Number of Transformer layers	N/A	3
Attention heads (nhead)	N/A	8
Feed-forward dimension	N/A	128 = 4 × d_model
Positional encoding	N/A	Sinusoidal (sin/cos; base 10,000; max_seq_len = 5000)
Activation function	N/A	GELU
Dropout	N/A	0.10
Normalization	N/A	LayerNorm (ε = 1e−5)
Output layer	N/A	Linear → Softmax (4 classes)
Weight initialization	He (Kaiming, for MLP hidden) / Xavier (Transformer layers) / Normal(0, 0.02) for output	
Loss function	N/A	Cross-Entropy loss

Training Details

Parameter	BYOL (Self-supervised)	Transformer-MLP (Classification)
Framework	PyTorch 2.1.2	PyTorch 2.1.2
Augmentation probability	0.5	0.3
Batch accumulation	1	1
Warm-up percentage	10 %	5 %
Checkpoints saved on	best validation loss	best validation loss
Validation patience	N/A	5 epochs
Final model weights	average of last EMA	best validation epoch (epoch 10)

Common Training Augmentations

All training methods employed the following image transformations:

- Random horizontal flip
- Random vertical flip
- Random sharpness (factor = 2)
- Gaussian blur (kernel = 5, σ = 1)
- Gaussian noise
- Random autocontrast
- Random solarize (threshold = 0.2)
- Random erasing
- Random affine (degrees = 10, translate = [0.1, 0.3])
- Resize (size = 300, antialias = True)
- Random resized crop (size = 300, antialias = True)

Input normalization: Per-channel min–max normalization was applied to all images. The same normalization procedure was identically applied to all external datasets.

Calibration and Deployment Parameters

Parameter	Value
Calibration method	Platt scaling (one-vs-rest, fitted on training set)
Operating threshold (per class)	Empirical 0.5 (after calibration)
Confidence threshold (τ)	0.77 (mean of correct and incorrect cases weighted by patch count on test cohort)
Low-confidence policy	Patient confidence < τ → re-imaging or human review
Failure rate	0 / 142 slide inferences failed
Primary evaluation metric	Macro-avg. balanced accuracy (patient-level)

Supplementary Figures

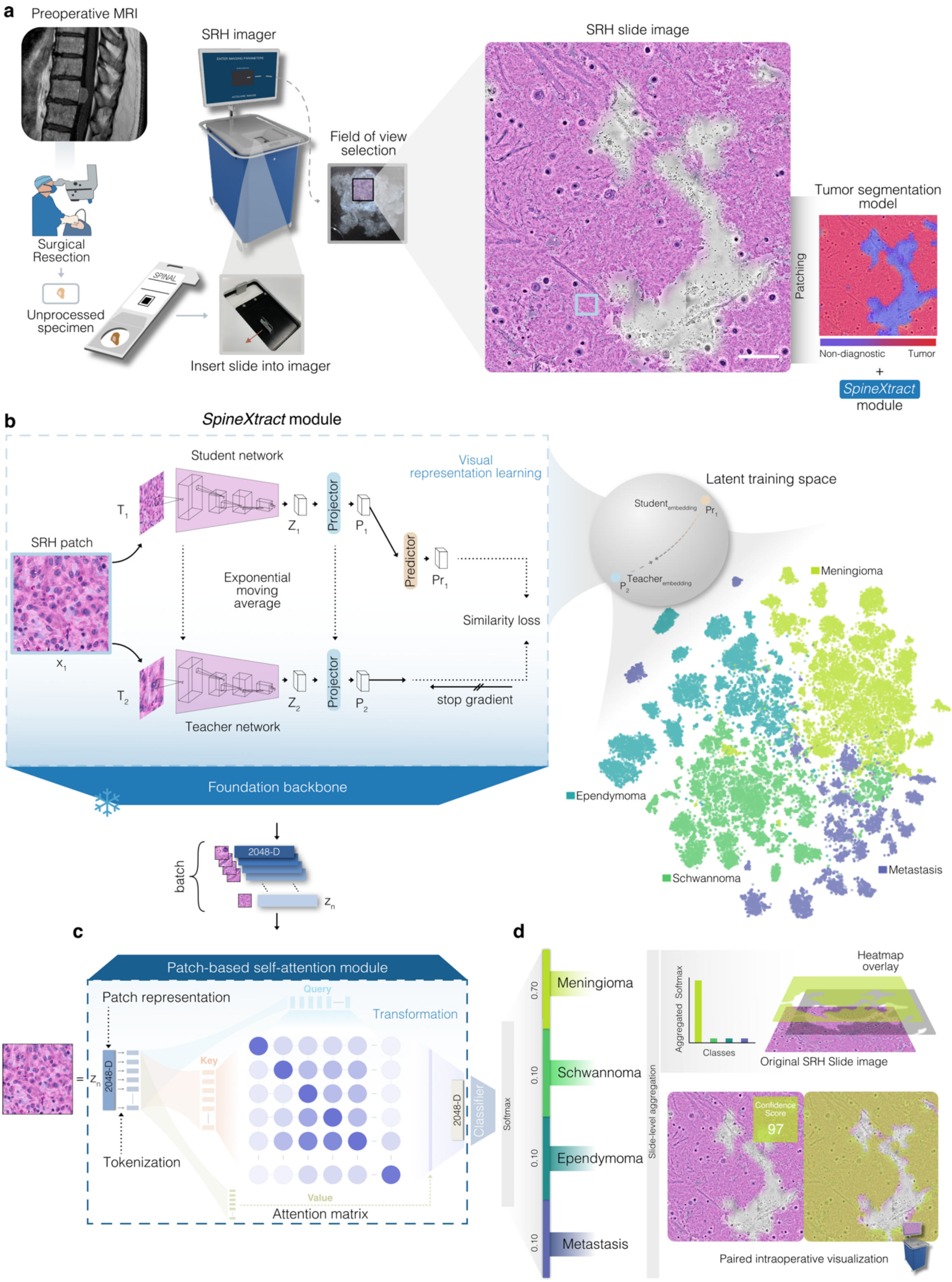

Supplementary Figure 1. The figure illustrates the SpineXtract system for near real-time tissue analysis in spinal tumor surgery. (a) Shows the workflow from preoperative MRI for indication surgery through surgical resection to processing tissue specimens in the SRH imager, resulting in SRH slide images that undergo tumor segmentation. (b) Depicts the SpineXtract module architecture with student-teacher neural networks processing SRH patches through representation learning pathways, creating a latent training space that differentiates tumor types like meningioma, ependymoma, schwannoma, and metastasis. (c) Presents a patch-based self-attention module that processes tokenized portions of the patch embedding vector through an attention matrix. (d) Displays classification results with confidence

scores and heatmap overlays on original SRH slide-images, providing immediate intraoperative visualization and model interpretability to guide surgeons and pathologists without processing delays.

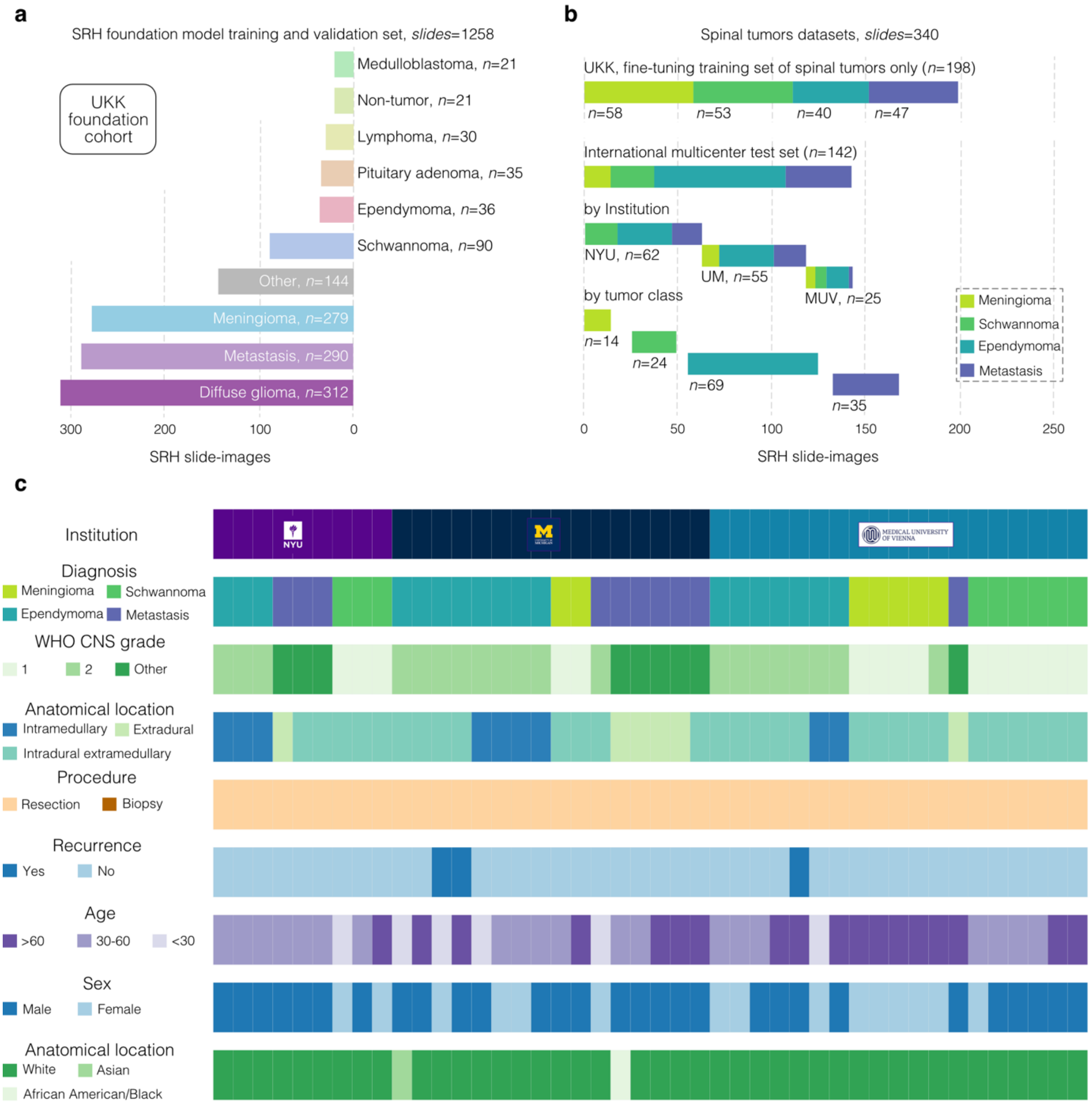

Supplementary Figure 2. This figure displays the datasets used for the SpineXtract model development pipeline. (a) Shows the SRH foundation model training and validation set comprising 1,258 slides from the UKK foundation cohort, featuring various tumor types including diffuse glioma (n=312), metastasis (n=290), meningioma (n=279), and other categories with corresponding SRH image counts. (b) Illustrates the spine tumor surgery datasets with 340 slides, divided into a UKK fine-tuning training set (n=198) and an international prospective multicenter test set (n=142), further categorized by institution (NYU, UM, MUV) and tumor class (meningioma, schwannoma, ependymoma, metastasis). (c) Presents the clinical and demographic characteristics of the prospective testing cohort, including institution, diagnosis, WHO CNS grade, anatomical location, procedure type, recurrence status, age, sex, and patient demographics, with each variable visualized through color-coded bars. Each bar represents one patient (n=44).

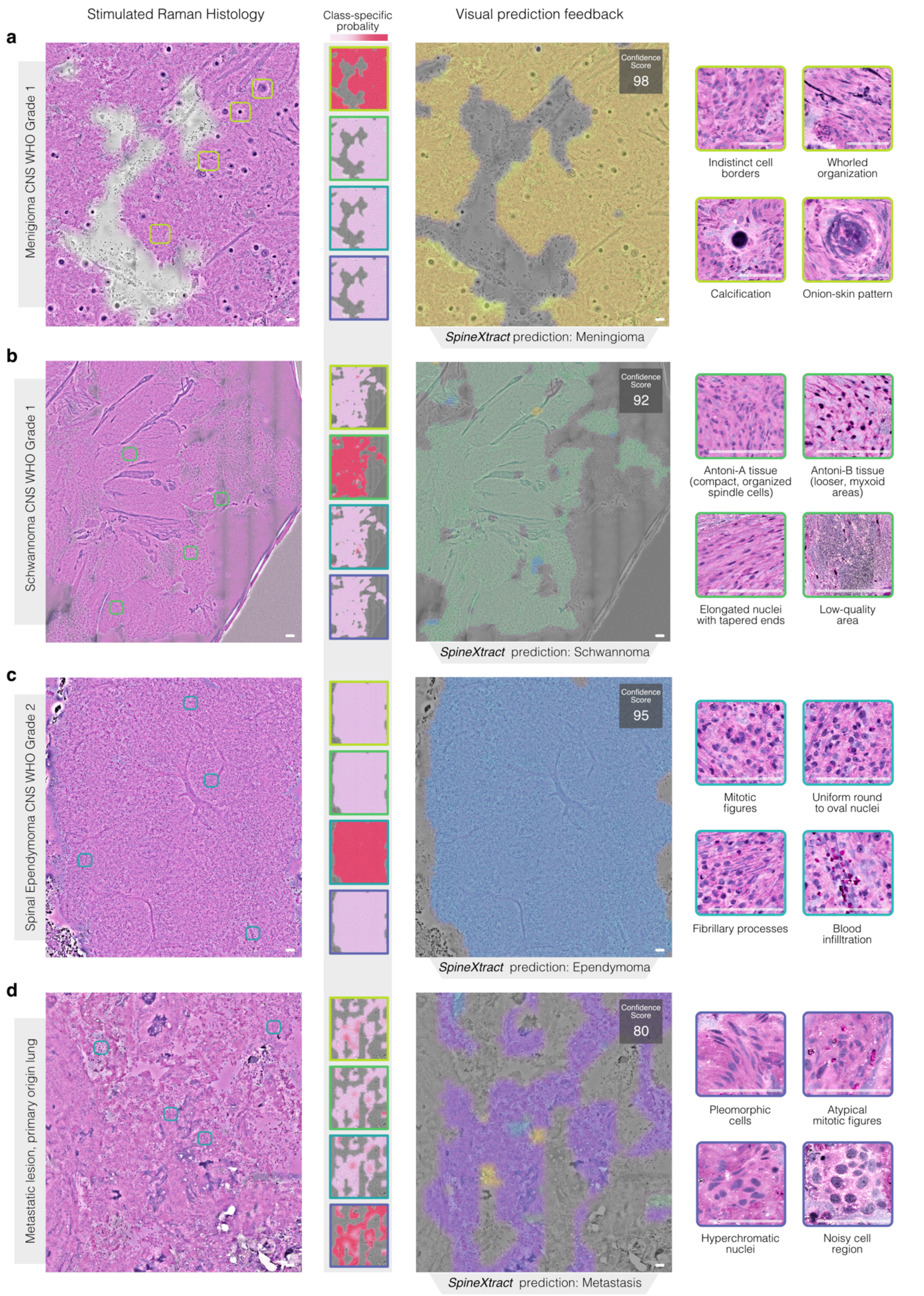

Supplementary Figure 3. SpineXtract's visual prediction feedback for intraoperative histopathological guidance and model interpretation. (a) Shows meningioma CNS WHO Grade 1 with its stimulated Raman histology slide-image, class-specific probability heatmaps, the model's visual prediction (confidence score 100), and characteristic histological features including indistinct cell borders, whorled

organization, calcification, and onion-skin pattern. (b) Displays schwannoma CNS WHO Grade 1 with its prediction (confidence score 98) and distinctive features such as Antoni A tissue (compact, organized spindle cells), Antoni B tissue (loose, myxoid areas), elongated nuclei with tapered ends, and a low-quality area. (c) Illustrates spinal ependymoma CNS WHO Grade 2 with high confidence score (98) and characteristic features including mitotic figures, uniform round to oval nuclei, fibrillary processes, and blood infiltration. (d) Shows metastatic lesion of primary lung origin with high confidence prediction (score 80) and typical features including pleomorphic cells, atypical mitotic figures, hyperchromatic nuclei, and noisy cell regions. Each row presents the original SRH image, class probability heatmaps, the model's prediction visualization, and highlighted histopathological features that aid in surgical decision-making.

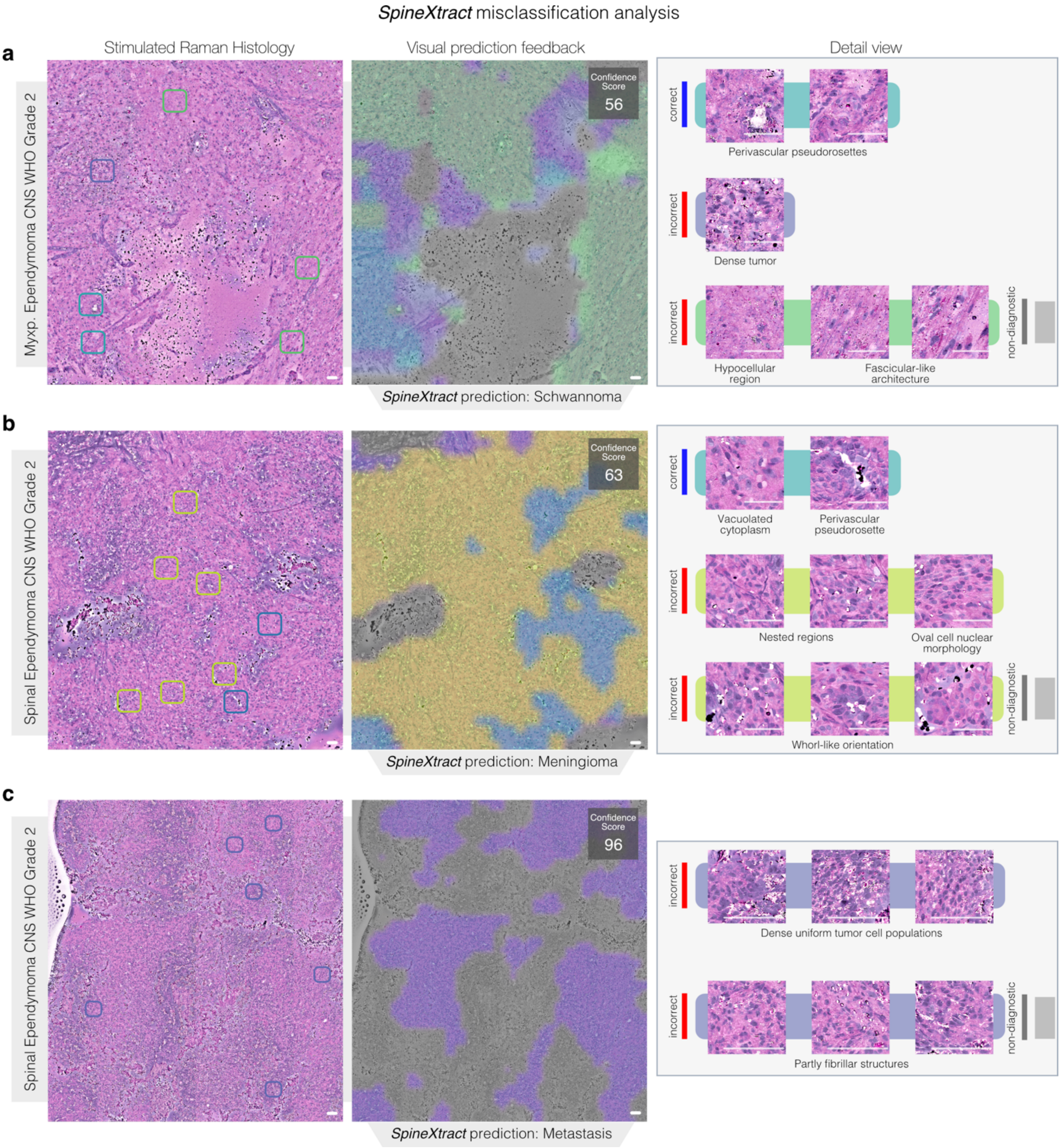

Supplementary Figure 4. SpineXtract misclassification analysis. Each row presents the original SRH slide-image, class probability heatmaps, the model's prediction visualization, and detailed histopathological features from misclassified regions. (a) Shows myxopapillary ependymoma CNS WHO

Grade 2 misclassified as schwannoma with confidence score 56. The detailed morphological analysis reveals overlapping features, including dense tumor regions, hypocellular areas, and spindle cell-like orientation that contributed to the classification error. (b) Displays spinal ependymoma CNS WHO Grade 2 incorrectly predicted as meningioma with confidence score 63, highlighting problematic histomorphological convergence, including nested regions, oval cell nuclear morphology, and whorl-like architecture that mimics meningothelial features. (c) Illustrates another spinal ependymoma CNS WHO Grade 2 misidentified as metastasis with perfect confidence score 96, showing dense, uniform tumor cell populations.

Calibration reliability and decision curve analysis - Multicenter testing cohort (all levels)

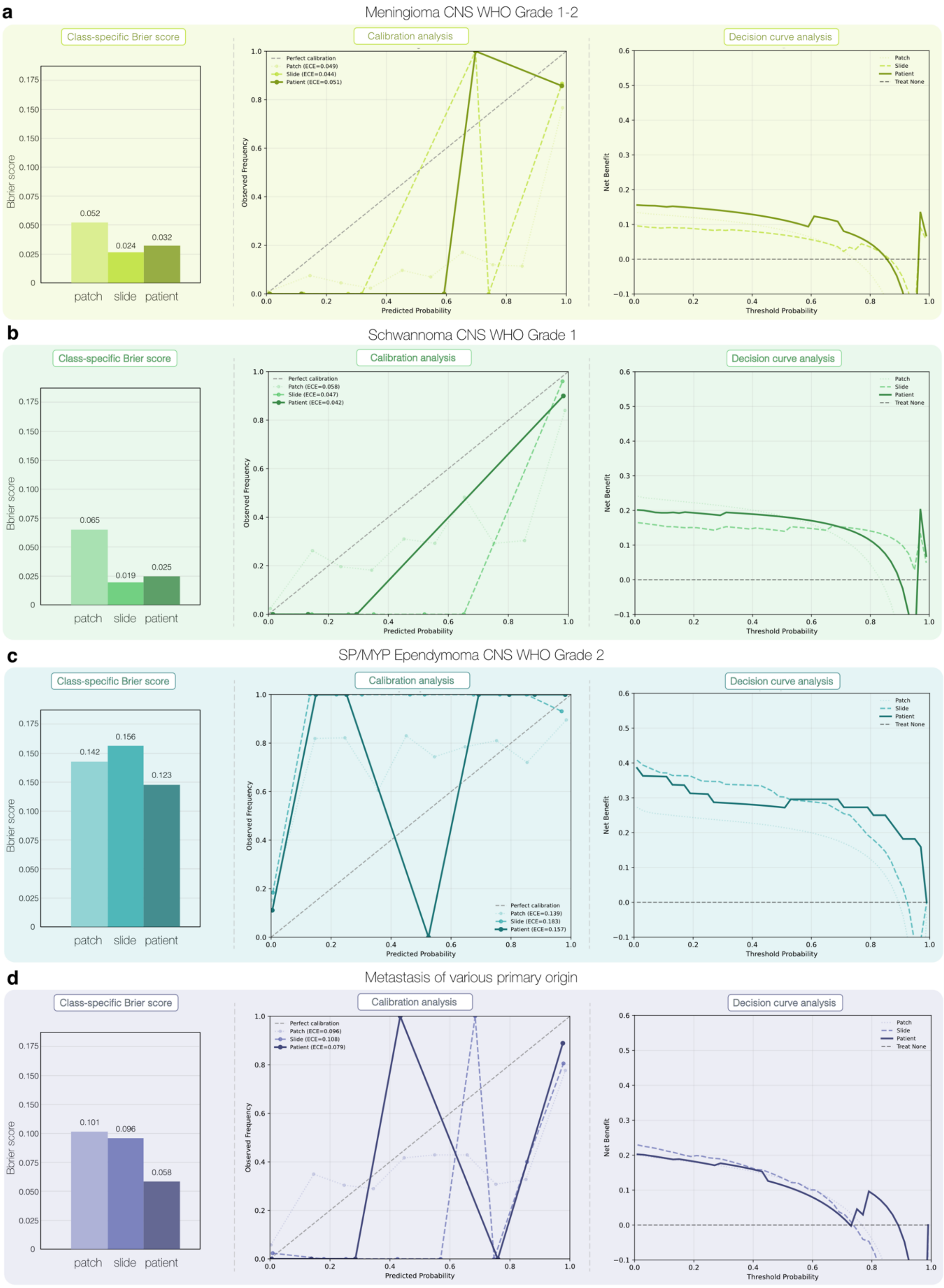

Supplementary Figure 5. Calibration reliability and decision curve analysis for multicenter testing cohort. Panels show class-specific calibration performance and clinical decision curve analysis for all four diagnostic entities at patch-, slide-, and patient-level predictions. (a) Meningioma CNS WHO Grade 1–

2 demonstrates excellent calibration with minimal deviation from the ideal line and the lowest Brier scores across all levels. (b) Schwannoma CNS WHO Grade 1 shows stable reliability with minor underconfidence at intermediate probabilities. (c) SP/MYP Ependymoma CNS WHO Grade 2 reveals wider variability and lower calibration, reflected by higher Brier scores and fluctuating observed frequencies. (d) Metastases of various primary origins display moderate calibration with a tendency toward overconfidence at high predicted probabilities. Decision curve analyses illustrate consistent net clinical benefit across a wide range of threshold probabilities, particularly at the patient level, supporting

the robustness of SpineXtract's calibrated predictions across tumor types.

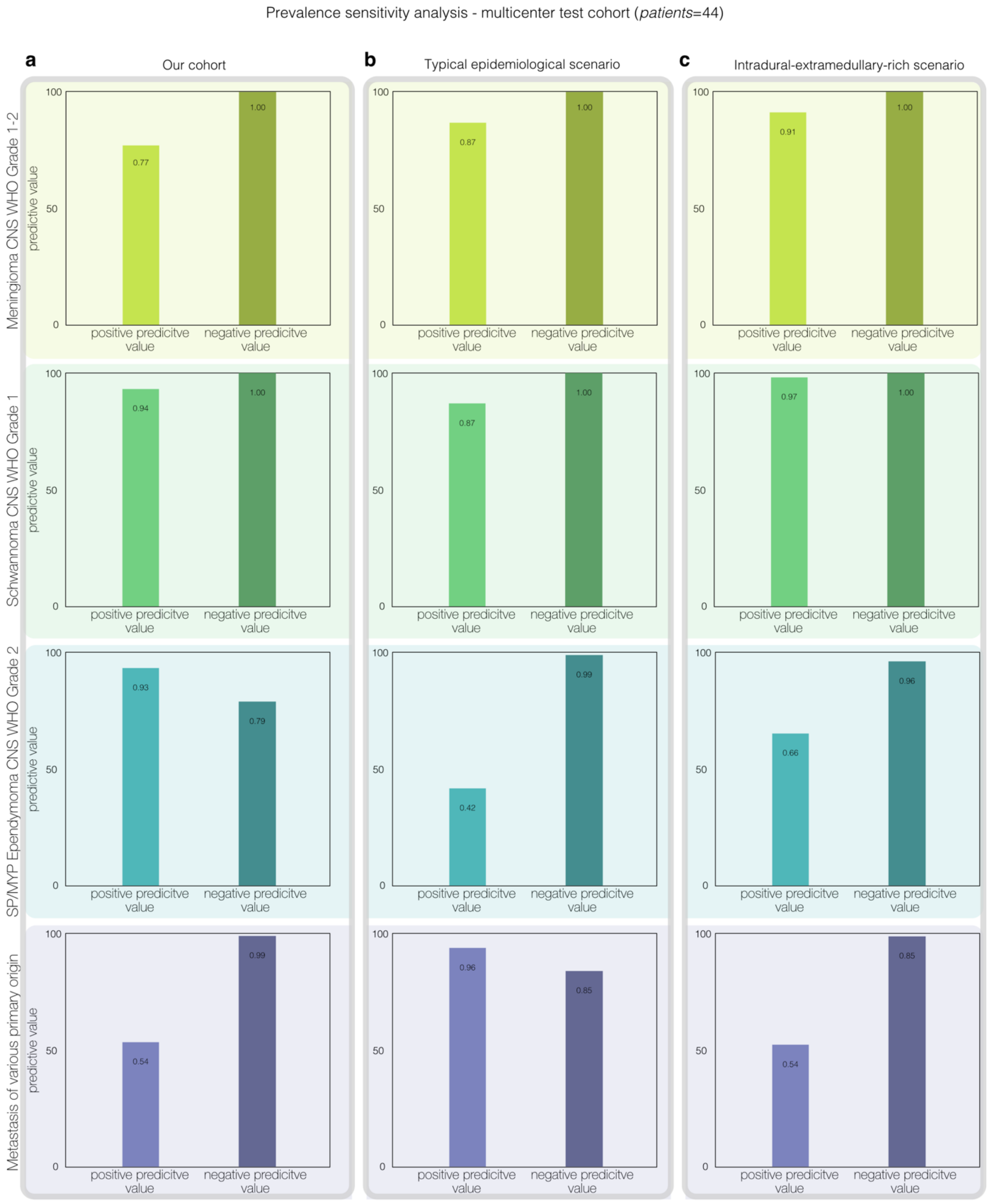

Supplementary Figure 6. Prevalence sensitivity analysis across different epidemiological scenarios. Panels display class-specific positive and negative predictive values (PPV and NPV) for all four tumor entities under varying prevalence assumptions in the multicenter testing cohort (n = 44 patients). (a) Our cohort represents the true observed case distribution in the study, showing consistently high NPVs across all tumor types. (b) Typical epidemiological scenario simulates expected real-world prevalence of spinal tumors, demonstrating maintained high NPVs but reduced PPVs for rarer entities. (c)

Intradural–extramedullary–rich scenario models a prevalence skew toward meningioma and schwannoma, resulting in improved PPV stability for these classes. Across all scenarios, the analysis demonstrates SpineXtract's robustness to prevalence shifts and its consistent diagnostic reliability across clinically different tumor distributions.

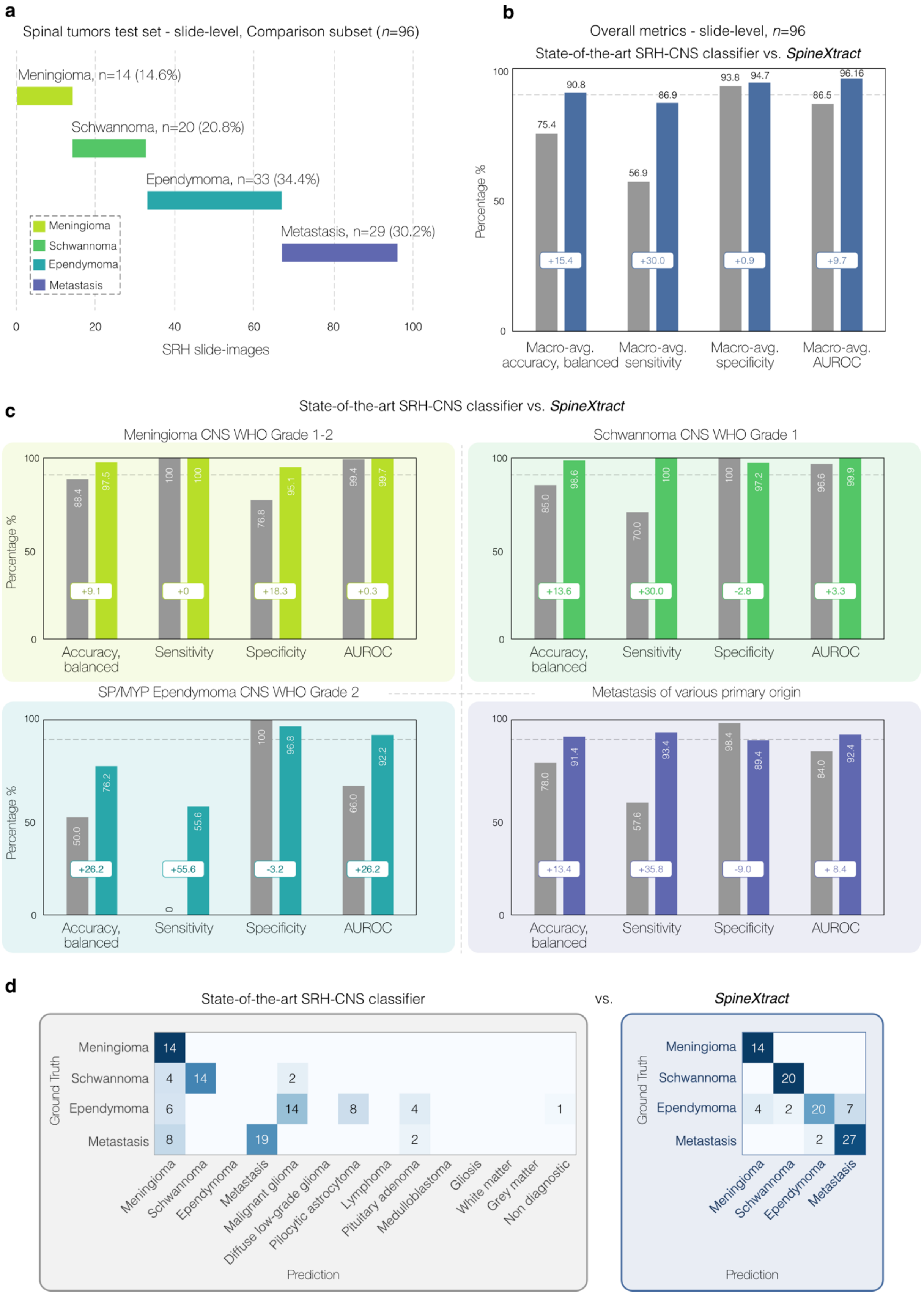

Supplementary Figure 7. Performance benchmarking at the slide level comparing SpineXtract with the state-of-the-art SRH CNS classifier. (a) Shows the spinal tumors slide-level test cohort composition (n = 96) used for benchmarking, with ependymoma representing the largest proportion, followed by metastasis, schwannoma, and meningioma. (b) Summarizes overall slide-level performance metrics across the cohort, where SpineXtract (blue bars) consistently outperforms the general SRH CNS classifier (gray bars) across all major evaluation criteria. (c) Depicts tumor-specific performance comparisons, demonstrating robust improvements for each diagnostic category and confirming the benefit of anatomically specialized training. (d) Confusion matrices illustrate SpineXtract's superior classification precision with markedly fewer inter-class confusions compared to the general classifier, reinforcing the findings from the patient-level ablation study that site-specific models achieve greater reliability and diagnostic discrimination in spinal tumor classification.

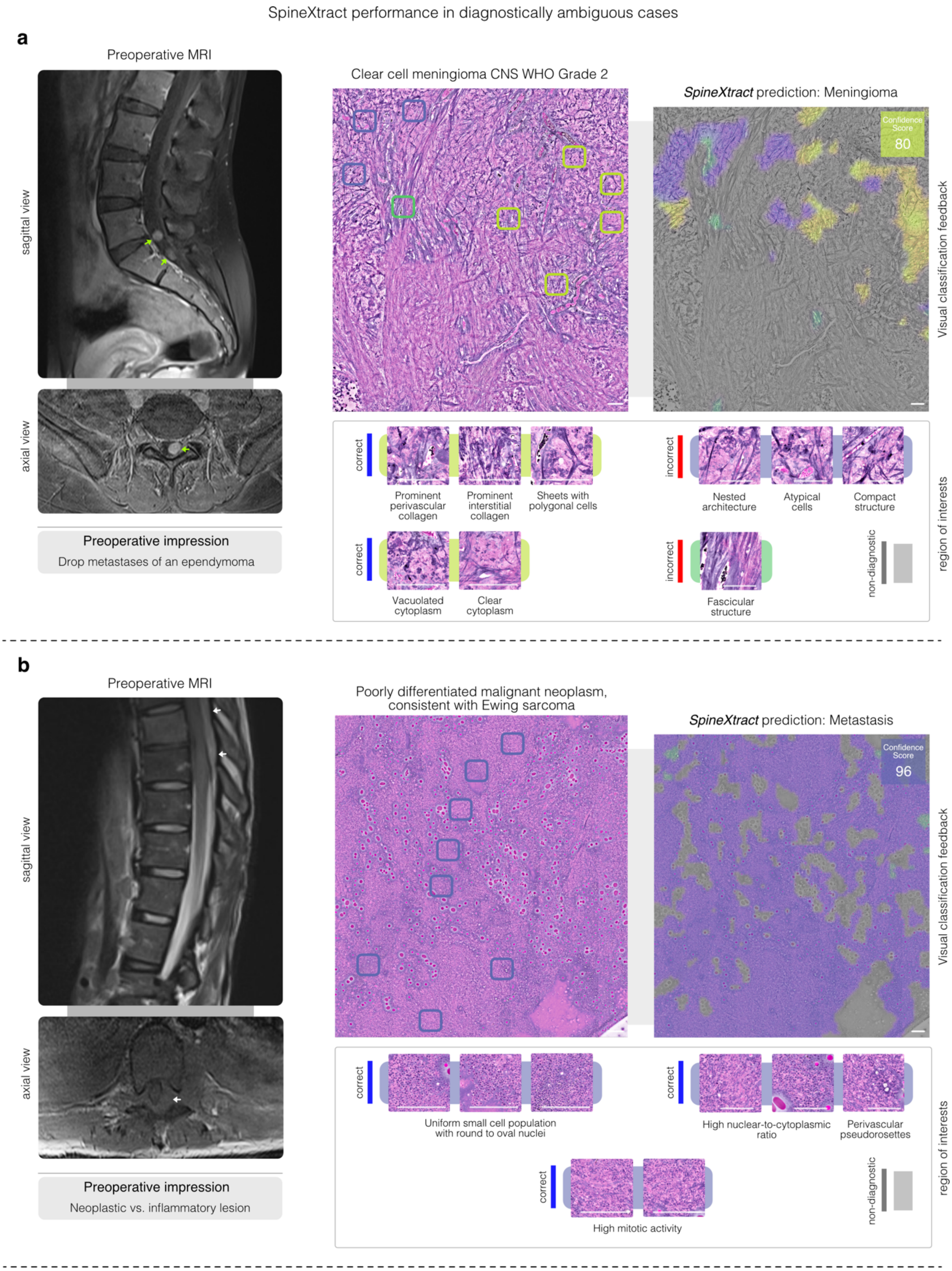

Supplementary Figure 8. SpineXtract performance of challenging spinal neoplasms. (a) Rare case of clear cell spinal meningioma (CNS WHO Grade 2) with initial clinical suspicion of ependymoma drop metastases in an adolescent woman. The preoperative MRI and the intraoperative surgeon's observation demonstrated lumbar and sacral intradural space-occupying lesions adherent to spinal rootlets, unusual for meningioma behavior. Final key histological features included clear cell

morphology, prominent interstitial collagen, and pleomorphic nuclei, consistent with clear cell meningioma diagnosis. Epigenetic DNA methylation profiling (Heidelberg CNS Tumor Methylation Classifier, DKFZ Brain classifier v12.8)[1] failed subtyping. SpineXtract correctly predicted meningioma (80% confidence) despite this rare clear cell subtype being absent from training data, demonstrating zero-shot generalization to unseen pathological variants. (b) Poorly differentiated malignant neoplasm consistent with Ewing sarcoma, initially suspected as unclear inflammatory versus neoplastic lesion. MRI showed thoracic epidural mass with paravertebral extension. Given persistent intraoperative uncertainty from both surgical and radiological perspectives, conventional frozen section was performed, which favored lymphoma. Final histology revealed uniform small round cells with a high nuclear-to-cytoplasmic ratio. FLI-1 labeling on IHC supported the diagnosis, favoring a secondary manifestation of Ewing sarcoma. SpineXtract demonstrated zero-shot generalization, accurately predicting metastasis of this rare mesenchymal primary with 96% confidence and correctly identifying characteristic features in this rare spinal presentation, despite not having encountered this entity during model training.

Supplementary Data S1 (Excel spreadsheet). This file provides de-identified patient- and slide-level data used in the study, including model predictions, per-class probabilities, and an overall confidence score, together with demographic and clinical variables. Each row represents a unique slide; identifiers are pseudonymous.

References

1. Capper D, Jones DTW, Sill M, et al. DNA methylation-based classification of central nervous system tumours. *Nature*. 2018;555(7697):469-474. doi:10.1038/nature26000

STARD-AI and TRIPOD-AI Checklists

STARD-AI (AI Diagnostic Accuracy Studies)

☒ Clearly identify the study as an AI diagnostic accuracy evaluation in the title or abstract.

☒ Describe the clinical setting and intended use of the AI tool.

☒ Define the target population, inclusion/exclusion criteria, and recruitment process.

☒ Describe the index test (AI model) and reference standard in detail.

☒ Specify how the AI model was developed/trained (data sources, architecture, hyperparameters).

☒ Describe the test dataset(s): number of patients, inclusion/exclusion, and site distribution.

☒ Ensure patient-level data partitioning and no overlap between datasets.

☒ Provide characteristics of the test population and differences from the training cohort.

☒ Detail preprocessing steps, augmentations, hardware/software used, and site-agnostic setup.

☒ Report performance metrics (sensitivity, specificity, AUC, PPV, NPV) with confidence intervals.

☒ Describe handling of missing data and outliers.

☒ Report measures to mitigate bias (e.g., blinding, independent test set).

☒ Discuss generalisability and external validation across institutions.

☒ Provide code, model weights, and technical specifications for reproducibility.

☒ Disclose funding sources, conflicts of interest, and ethics approval.

TRIPOD-AI (Prediction Model Studies Using AI/ML)

☒ Title and abstract: identify model type, outcome, and target population.

☒ Provide background, clinical context, and rationale for the model.

☒ Describe data sources, settings, and time periods.

☒ List eligibility criteria and recruitment process for participants.

☒ Define outcomes and predictors with details on measurements and blinding.

☒ Justify sample size and describe handling of missing data and outliers.

☒ Describe model type, feature selection, and hyperparameter tuning.

☒ Explain data split strategy (development, validation, external test).

☒ List performance metrics and how uncertainty was quantified.

☒ Include participant flow diagram and baseline characteristics.

☒ Present final model specification (predictors, coefficients/weights).

- ☒ Report discrimination, calibration, and external validation results.
- ☒ Discuss results in context of prior studies, strengths, and limitations.
- ☒ Discuss fairness, equity, and generalisability of the model.
- ☒ Provide access to code, model, data (if possible), and ethics details.
